# Understanding the role of interactions between host and *Mycobacterium tuberculosis* under hypoxic condition: an *in silico* approach

**DOI:** 10.1186/s12864-018-4947-8

**Published:** 2018-07-27

**Authors:** Tungadri Bose, Chandrani Das, Anirban Dutta, Vishnuvardhan Mahamkali, Sudipta Sadhu, Sharmila S. Mande

**Affiliations:** 1 Bio-Sciences R&D Division, TCS Research, Tata Consultancy Services Limited, Pune, India; 2 Department of Chemical Engineering, Indian Institute of Technology Bombay, Mumbai, India; 3 Present Address: Australian Institute for Bioengineering and Nanotechnology (AIBN), The University of Queensland, Brisbane, Australia

**Keywords:** *Mycobacterium tuberculosis* infection, Hypoxia, Host-pathogen interactions, Gene regulatory network, Genome scale metabolic model, Flux balance analysis

## Abstract

**Background:**

*Mycobacterium tuberculosis* infection in humans is often associated with extended period of latency. To adapt to the hostile hypoxic environment inside a macrophage, *M. tuberculosis* cells undergo several physiological and metabolic changes. Previous studies have mostly focused on inspecting individual facets of this complex process. In order to gain deeper insights into the infection process and to understand the coordination among different regulatory/ metabolic pathways in the pathogen, the current *in silico* study investigates three aspects, namely, (i) host-pathogen interactions (HPIs) between human and *M. tuberculosis* proteins, (ii) gene regulatory network pertaining to adaptation of *M. tuberculosis* to hypoxia and (iii) alterations in *M. tuberculosis* metabolism under hypoxic condition. Subsequently, cross-talks between these components have been probed to evaluate possible gene-regulatory events as well as HPIs which are likely to drive metabolic changes during pathogen’s adaptation to the intra-host hypoxic environment.

**Results:**

The newly identified HPIs suggest the pathogen’s ability to subvert host mediated reactive oxygen intermediates/ reactive nitrogen intermediates (ROI/ RNI) stress as well as their potential role in modulating host cell cycle and cytoskeleton structure. The results also indicate a significantly pronounced effect of HPIs on hypoxic metabolism of *M. tuberculosis*. Findings from the current study underscore the necessity of investigating the infection process from a systems-level perspective incorporating different facets of intra-cellular survival of the pathogen.

**Conclusions:**

The comprehensive host-pathogen interaction network, a Boolean model of *M. tuberculosis* H37Rv (Mtb) hypoxic gene-regulation, as well as a genome scale metabolic model of Mtb, built for this study are expected to be useful resources for future studies on tuberculosis infection.

**Electronic supplementary material:**

The online version of this article (10.1186/s12864-018-4947-8) contains supplementary material, which is available to authorized users.

## Background

Invasion by pathogens is known to trigger an array of immune responses in the host. Pathogens, in turn, try to counter-act, subvert, or alter these responses [1, 2]. The progression through different stages of infection therefore involves an array of complex interactions between the host and the pathogen proteins [3]. Virulent strains of *Mycobacterium tuberculosis* can evade the immune response and remain in a dormant (or latent) stage within the host cell for prolonged periods (often for several years), without causing any symptoms before reactivation [4, 5]. To adapt to the ‘hostile’ intra-cellular environment of the host, the cells of *M. tuberculosis* undergo several physiological and metabolic changes [6]. An elaborate response mechanism of *M. tuberculosis* against hypoxic (low oxygen) conditions is one of the primary features characterizing its state during latency [7–9]. Mycobacterial proteins involved in host-pathogen interactions are likely to play crucial roles in sensing the host environment and subsequently participate (directly or indirectly) in regulatory events, leading to physiological/ metabolic changes necessary for intra-cellular survival. Understanding of the *M. tuberculosis* survival strategies, including changes in gene regulatory cascades as well as metabolism, would therefore remain incomplete unless studied in context of the host-pathogen interactions.

The following aspects pertaining to intra-cellular hypoxic survival of *M. tuberculosis* inside human macrophage cells have been investigated in this work. (I) A comprehensive host-pathogen interactome network, depicting the potential interactions between an invading *M. tuberculosis* cell and a human macrophage, has been built. (II) A Boolean model of the gene regulatory network controlling hypoxic response in *M. tuberculosis* has been constructed and simulated to understand the progression of hypoxia and its downstream regulatory effects. (III) The metabolic changes experienced by a *M. tuberculosis* cell during hypoxic conditions have been simulated using FBA (Flux Balance Analysis) models of *M. tuberculosis* metabolism. Subsequently, the cross-talks between these aspects have been investigated to understand how the cellular and metabolic changes are driven by the gene regulatory events as well as the host-pathogen interactions.

## Results

### Host-pathogen interactions (HPI) between human and *M. tuberculosis* H37Rv (Mtb)

**Fig. 1:**
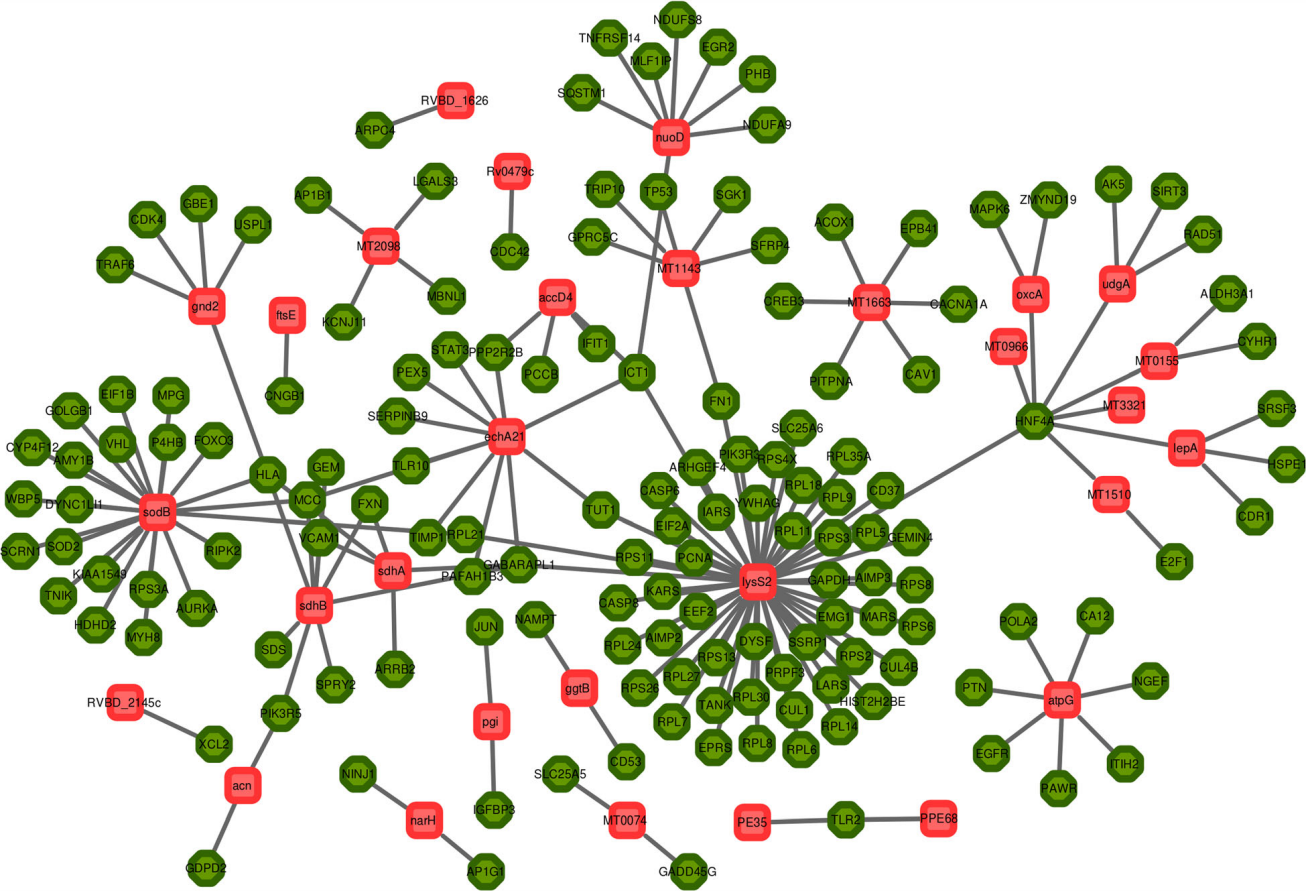
Host-Pathogen Interaction (HPI) network. The constructed HPI network consisted of 174 interactions (edges) involving 148 human proteins (green) and 30 *M. tuberculosis* H37Rv (Mtb) proteins (red)

A template protein-protein interaction (PPI) library was constructed by collating PPI related information from different public resources and potential HPIs between human and Mtb proteins was obtained by interlogs mapping approach (described in Additional file 1). The obtained set of HPIs was further filtered based on the following information curated from literature - (a) cellular localization of the participating proteins, and (b) gene expression profiles of the invading pathogen as well as that of the infected host cells. The final set of HPIs comprised of 178 intra-species PPIs involving 148 human and 30 Mtb proteins (Fig. 1) (also see described in Additional file 2). The human and Mtb proteins participating in HPIs were also observed to be well connected with each other in the respective intra-species PPI networks (background networks). The human-Mtb HPI network, augmented with such intra-species interactions, comprised of 178 nodes and 391 edges (data not shown). A single large sub-network consisting of a majority of the proteins participating in the predicted HPIs (142 out of 148 human proteins and 24 out of 30 Mtb proteins), and five small sub-networks, each consisting of two or three nodes (proteins), could be identified from this HPI network. Similar network architecture of human-Mtb HPIs has also been reported earlier [10]. Interestingly, the average degree of the constructed network (4.4) was found to be comparable to that of the organism specific (human as well as Mtb) PPI networks.

The role of a number of the predicted interactions in host immunity and bacterial pathogenesis could be validated from previously published literature (details in Additional file 2). For example, the predicted interaction between the human protein CDC42 and bacterial Mtb protein CipA/ Rv0479c (both up-regulated during infection – see Additional file 3) assumes importance given to the role of CDC42 in cytoskeletal rearrangements of the host cell. Cytoskeletal rearrangement of the host cells during phagocytic uptake of invading pathogens has been reported in earlier studies [11, 12]. Another predicted HPI involving human nicotinamide phosphoribosyltransferase (Visfatin) and the Mtb protein gamma-glutamyltransferase (GGT/Rv2394) could be linked to a possible defensive strategy of the pathogen against increased cellular levels of glutathione (GSH). GSH on cleavage by GGT forms a dipeptide [Cys(NO)-Gly] which has bactericidal effects [13]. While upregulation of Visfatin during infection may induce higher cellular levels of GSH [14], downregulation of GGT by Mtb is expected to play a protective role (see Additional file 3). Among others, HPI pertaining to human protein ninjurin (NINJ1) and nitrate reductase (NarH) of Mtb may be noted for its probable role in reducing the nitrate mediated stress exerted by the host cell through ninjurin activity [15]. The superoxide dismutase protein (SodA) of Mtb was predicted to interact with a number of human proteins, re-emphasizing the role of SodA in Mtb pathogenecity. In particular, HPI involving human HLA class I histocompatibility antigen and the Mtb SodA indicated the possibility of the presence of a HLA specific epitopic region within the mycobacterial SodA. This notion is also supported by existing literature [16] and assumes importance in antigen presentation for triggering host immune responses.

The role of the human proteins (associated to the identified host-pathogen interactome) were investigated in context of the KEGG pathways [17], in order to gauge the effect of Mtb infection on host metabolic/ functional pathways (details in Additional file 4). Most of the interacting host proteins (89 out of 148 human proteins involved in HPIs) were seen to be associated with the KEGG Tuberculosis pathway (ko05152). In addition, host mechanisms associated with metabolic or biological functions like apoptosis, Toll-like receptor signalling, NOD-like receptor signalling, MAPK signalling, production of pro-inflamatory cytokines and Type-I inteferons were seen to be perturbed during Mtb infection.

### Gene regulatory network (GRN) controlling hypoxic response in *M. tuberculosis* H37Rv (Mtb)

A set of 286 genes (Additional file 5), encompassing the *dosR* regulon and enduring hypoxic response (EHR), was identified to be involved in hypoxic response through literature mining and were integrated into a comprehensive gene regulatory network. The role of transcription factors (TF) network which orchestrates the gene regulatory events during Mtb hypoxic response was analysed using a multi-level Boolean modelling (details in Additional files 6 and 7). A model was constructed for the network comprising of 24 TFs belonging to dosR and EHR regulons. Simulations of the model were performed using an initial state which mimicked the biological state (gene expression levels) at the beginning of hypoxic stress. For over 70% of the total TFs (nodes) in the network, the stable state values obtained from simulation were observed to be in coherence with the experimental values (Additional file 8). Apart from capturing the gradual changes in pattern of expression for most of the TFs, the model could replicate the oscillatory gene expression patterns of the two TFs (*kstR* and Rv0494) which were observed in an earlier reported study [8] (Additional files 8 and 9).

The constructed Boolean model was further utilized to predict the key regulators that are essential for the survival of Mtb in the host intra-cellular environment. For this purpose, the downstream target genes corresponding to each of the 24 TFs were appended to the network (details in Additional files 6 and 7). An analysis of the topological properties of this extended network revealed a number of high degree nodes, such as DosR, Rv0081, Lsr2, CsoR and Rv0324. Earlier studies have indicated DosR and Rv0081 to be important hub regulators of Mtb hypoxic response [7–9, 18, 19]. Lsr2 has also been proposed earlier to be a global transcriptional regulator, which responds to variations in oxygen level, and thus contributes to persistent infection [20]. It is also probable that the other two high degree proteins (CsoR and Rv0324), also act as hub regulators of hypoxic response in Mtb due to the following reasons. CsoR has been reported to be involved in copper homeostasis and plays a probable role in intra-cellular survival and virulence of the pathogen [21]. On the other hand, Rv0324 has been suggested to modulate cholesterol metabolism through enhancing the expression of another regulator KstR [22].

In addition to studying the static properties, the dynamic properties of the gene regulatory network (GRN) were also investigated through simulation of *in silico* mutant models (details in Additional files 6 and 7). Since the steady state obtained from the simulations represents the state of proteins in enduring (or late) hypoxic response, analyses of such states pertaining to the mutants are expected to help in deciphering importance of the TFs in sustaining the response at late stages of infection. Simulation studies (single gene knock-outs) revealed that mutations in seven out of the 24 TFs, affected the behaviour of downstream genes significantly, when studied in terms of enriched pathways (details in Additional files 6 and 7). These seven TFs included CsoR, Rv0081, Rv2034, Lsr2, PhoP, Rv1985c and Rv0324. *ΔcsoR* was observed to have the most prominent effect, altering the pathway enrichment pattern of downstream genes by as much as ~ 37% (when compared to the wild type). Interestingly, despite being a high degree TF with a known role in hypoxic response, the *dosR* mutant did not exhibit any appreciable changes in the pathway enrichment patterns, when compared to the wild type Mtb. This indicates that, although DosR plays a crucial role in initiating hypoxic response, its role in maintenance of bacteriostasis and enduring hypoxic response is probably not very significant. An earlier study had also indicated a nominal effect of *dosR* deletion on bacteriostasis under hypoxic environment [8]. On the other hand, the current observations further strengthens our hypothesis of CsoR being a hub regulator (of hypoxic response), influencing a number of pathways required by the bacteria to survive under intra-cellular stress at late stage of infection. In summary, the above observations suggest probable roles of CsoR and Rv0324 as key regulators of certain Mtb pathways which are required for adaptation to intra-cellular stress. However, a deeper probe would be required to understand the exact mechanisms of hypoxic regulation by these two TFs.

**Fig. 2:**
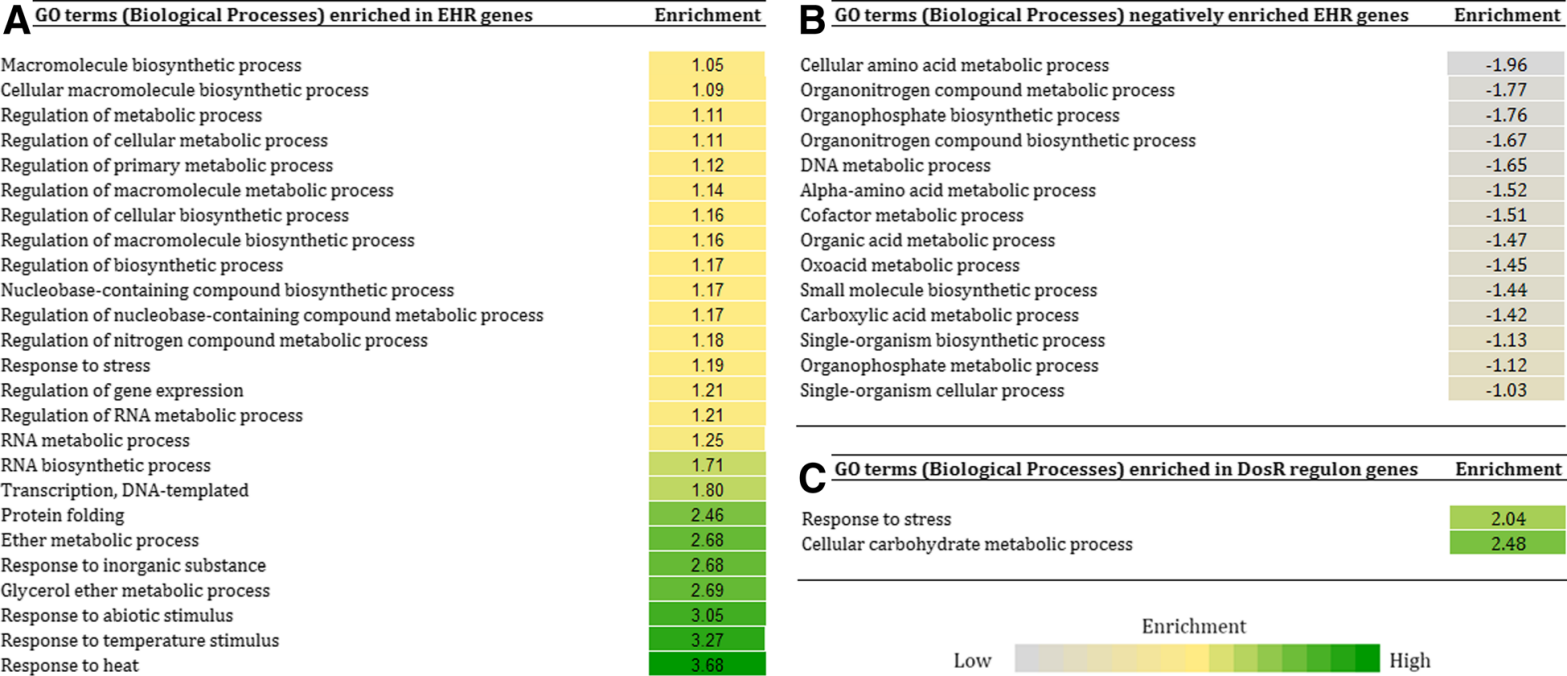
Pathways enriched in hypoxia associated genes of *M. tuberculosis* H37Rv (Mtb). Pathways (GO terms) in Mtb that are observed to be (**a**) enriched in genes corresponding to enduring hypoxic response (EHR), (**b**) negatively enriched in EHR genes and (**c**) enriched in dosR regulon genes involved in initial hypoxic response

Functional analysis of the genes encompassing the *dosR* regulon as well as enduring hypoxic response (EHR), provided further interesting insights into pathway level changes that are likely to occur in Mtb under hypoxic stress. Despite a large set of genes pertaining to both *dosR* regulon (33 out of 57) and enduring hypoxic response (EHR) (160 out of 230) remains unannotated till date, a majority of the ‘annotated’ set was observed to be involved either in metabolic processes or stress response pathways. A pathway enrichment analysis was performed in order to identify the up and down regulated pathways under hypoxic stress (Fig. 2). Apart from stress response, the pathways enriched during EHR mainly included ether and glycerol metabolism as well as regulation of metabolic process (Fig. 2a). This finding complements earlier literature, wherein lipid has been reported to be the preferred energy source for intra-cellular survival of pathogens [23]. On the other hand, the pathways observed to be negatively enriched during EHR were found to be related to metabolism of DNA and amino acids (Fig. 2b). Reduced metabolism of DNA and amino acids are likely to help the pathogen in controlling its growth, which in turn may aid in evading the host immune response and maintain dormancy [24].

The results of the analysis performed on the genes for initial hypoxic response further revealed enriched pathways related to stress response and carbohydrate metabolism. Considering the imminent slower growth phase the pathogen is likely to encounter in the subsequent EHR stage, enhanced carbohydrate metabolism is contrary to expectations. Thus exact functional roles of the genes contributing to the enriched carbohydrate metabolic process were investigated. Only two initial hypoxic response genes were observed to be associated to this process, namely, *otsB1* (Rv2006) and *glpQ1* (Rv3842c). OtsB1 has been suggested to act as a trehalose monomycolate phosphatase, which participates in the synthesis of a cell-wall component arabinogalactan-mycolate [25]. However, no role in Mtb metabolism has been suggested yet for GlpQ1, a putative glycerophosphoryl diester phosphodiesterase [26]. These observations suggest that the enrichment of carbohydrate metabolism during initial hypoxic response (indicated in our analysis) probably pertains to maintenance or synthesis of the cell wall components under intra-cellular stress, and may not signify an enhanced energy metabolism.

Pathways which were observed to be differentially ‘enriched’ during early and enduring hypoxic response hinted at the distinct responsibilities shouldered by *dosR* and EHR regulons during hypoxic adaptation. While the pathogen, when exposed to host intra-cellular environment, initially employs certain stress response mechanisms to counter the host immune system, later stages of hypoxia is additionally characterized by gene regulatory events leading to a slower (or arrested) growth and a preference for lipids as energy source.

### *M. tuberculosis* H37Rv (Mtb) metabolism under hypoxic conditions

The environment inside the macrophage is typically hypoxic and is characterized by low pH [24, 27]. Earlier studies have shown that Mtb undergoes a metabolic shift from carbohydrates to lipids as the preferred source of carbon for its viability during hypoxia [23, 28]. Observations made in the current analysis also suggested that genes corresponding to ether and glycerol metabolic processes could be enriched under hypoxic growth conditions (described in previous section). In order to obtain a deeper understanding of the effect of hypoxia on the metabolism of Mtb, we performed an *in silico* flux balance analysis (FBA) study (Additional files 10 and 11). As compared to the steady state, metabolic pathways constituting the central carbon metabolism (such as, TCA cycle, pyruvate metabolism and glycolysis) were seen to be perturbed during hypoxia (Additional files 10 and 12). Further, fluxes flowing through reactions of nucleotide metabolism (mostly purine metabolism) and amino acids metabolic pathways (pertaining to arginine, proline, glycine, serine and threonine, etc.) were found to be altered. In addition, redox reactions were also observed to carry differential flux under simulated normal and hypoxic conditions. The simulations could also reproduce the experimentally observed assimilation of CO_2_ through anaplerotic pathways [29, 30] leading to the production of oxaloacetate (OAA) under glucose limiting conditions.

**Fig. 3:**
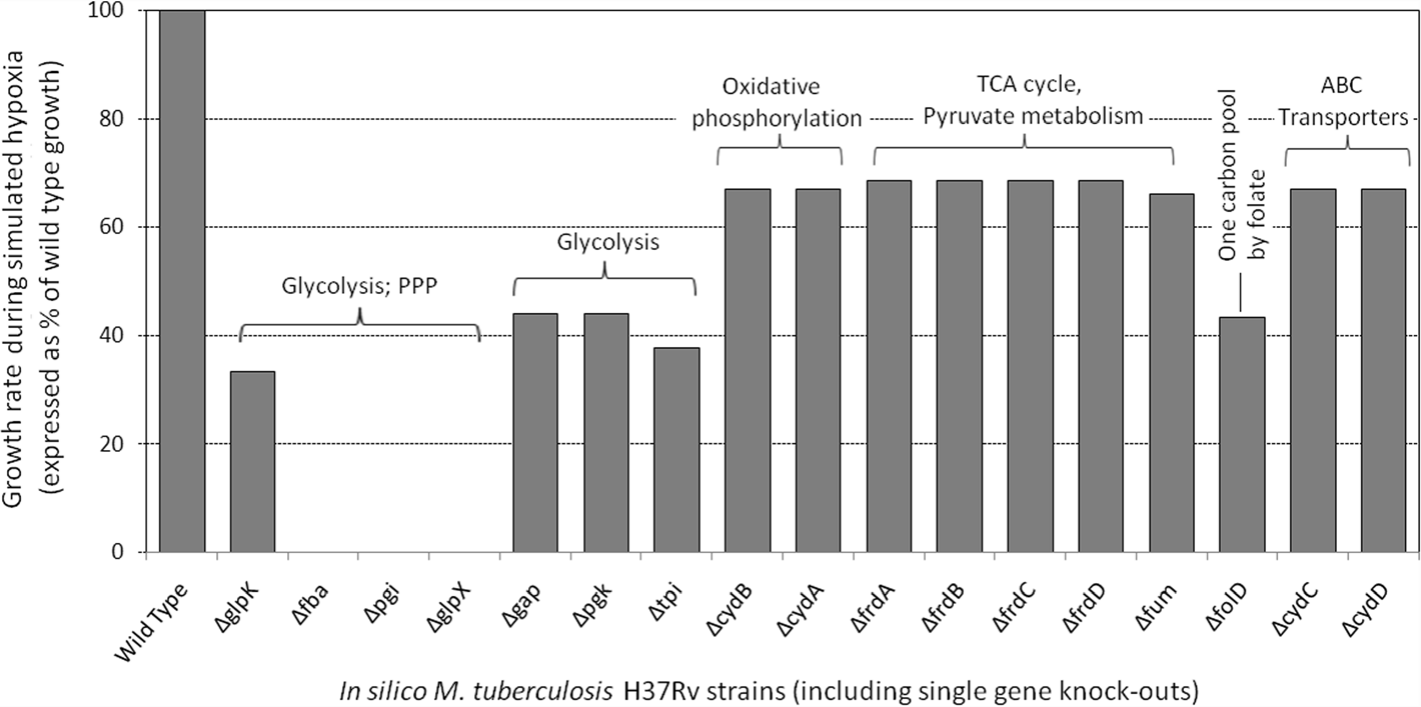
Comparative growth rates of different mutants of M. tuberculosis H37Rv (Mtb) compared to wild type bacilli. Relative simulated growth rates (obtained through FBA simulations mimicking hypoxia) of different in silico gene knock-outs of Mtb as compared to the wild type bacilli. Biological functions associated to the knocked-out genes are also indicated in the plot

In order to adapt to the host intra-cellular environment (especially under hypoxia), the invading Mtb is expected to undergo substantial changes in its metabolism. We made an attempt to identify the key enzymes which could be pivotal in bringing about these changes. A systematic gene knock-out simulation study under low oxygen conditions was performed using the iNJ661 FBA model after incorporating the missing reactions for cholesterol metabolism. Figure 3 shows the relative growth rates of the simulated Mtb bacilli when different single (*in silico*) gene-knock out studies was performed.

The growth of the simulated Mtb cell was seen to be most affected in the mutants lacking genes from the glycolysis or gluconeogenesis pathways (viz., *Δfba*, *Δpgi*, *ΔglpX*, *Δgap*, *Δpgk*, and *Δtpi* mutants). In particular, the bacterial cells failed to grow in the absence of *fba*, *pgi*, and *glpX.* It may be noted that these three enzymes, apart from contributing to glycolysis/ gluconeogenesis pathways, are also key components of the pentose phosphate shunt pathway (PPP) which has been reported to play an important role in Mtb survival under low pH conditions and oxidative stress [31]. The growth of the Mtb cell was also seen to be severely compromised in the *ΔglpK* and *ΔfolD* mutants. *glpK* encodes for glycerol kinase and is a key enzyme in the regulation of glycerol uptake and metabolism [32]. Our observation is in line with an earlier study reporting dysgonic growth of *ΔglpK* Mtb cells [33]. FolD, on the other hand, is a bi-functional enzyme and plays important role in intermediary metabolism and respiration, and has been reported to be essential for the survival of Mtb under in vitro hypoxic conditions [26]. The present result also suggests that *ΔfolD* mutants are severely compromised for growth under intra-cellular hypoxic conditions. In addition, mutations pertaining to enzymes from the TCA cycle, oxidative phosphorylation, and pyruvate metabolism were also seen to affect the growth of the Mtb cell to a certain degree. To summarize, certain enzymes corresponding to the PPP were found to be crucial for the survival of Mtb during hypoxia. Re-routing of metabolic flux through the PPP generates reducing equivalents (in the form of NADPH) which may help the Mtb cells to counter the oxidative stress asserted by the host during hypoxia.

### Cross-talk between *M. tuberculosis* H37Rv (Mtb) HPI network and gene regulatory network under hypoxic condition (hypoxic-GRN) with different cellular processes

#### *M. tuberculosis* H37Rv (Mtb) cellular processes associated with the HPI network

To probe whether the Mtb proteins participating in the predicted HPIs, as well as those involved in the hypoxic gene regulatory network (GRN), can influence other biological pathways/ processes, gene ontology (GO) enrichment analyses were performed (see Additional file 6). Additional file 13 lists the enriched GO terms identified when the set of mycobacterial proteins involved in HPIs, along with their 1st degree neighbours, were considered.

A majority of the enriched GO terms pertained to biological processes like ‘generation of precursor metabolites and energy’ and ‘carbohydrate catabolic process’. Amongst these, the glycolytic process (GO:0006096) was observed to be up-regulated only during the early time points of infection. It is likely that intermediates of carbohydrate catabolic process are utilized for generation of ATP during early stages of infection. However, since prolonged hypoxia-induced glycolysis may have toxic effects on mycobacteria [34], a subsequent reduction in glycolytic processes may be necessitated by the cell during later stages of infection. It may further be noted in this context, that the metabolic pathway enrichment study also indicated enrichment of carbohydrate metabolic processes during early phases of hypoxia (as discussed in an earlier section). This apparent enrichment of carbohydrate metabolic processes could be attributed to trehalose production, which contributes to cell wall synthesis.

Pentose-phosphate shunt pathway (GO:0006098) was also observed to be enriched during the early stages of infection. This pathway is known to provide reducing equivalents for reductive biosynthetic reactions in Mtb [28], and it’s up-regulation may play a possible role in balancing ROI/RNI stress induced by the host cell. It is interesting to note that the up-regulation of the pentose phosphate pathway was also not sustained by the myobacteria till late infection time points. Further analysis on the gene expression profiles of the infecting mycobacterial cell as well as that of the host phagocyte (macrophage) revealed that the regulation of pentose-phosphate pathway (in Mtb) was temporally correlated with the human respiratory burst mechanism (Additional files 2 and 3). Given that the human respiratory burst mechanism is responsible for inducing ROI/RNI stress [35], our observations indicate a fine balance between the host response to invasion and the self defence mechanisms of the pathogen.

The iron-sulfur cluster assembly process of Mtb (GO:0016226) was found to be up-regulated during all stages of infection. Given that vital cellular processes like electron transport, energy metabolism and DNA synthesis require iron-containing cofactors, this observation is in line with expectations. In addition, it is also likely that this up-regulation confers resistance to iron limitation and oxidative stress, as suggested in an earlier report [36].

#### Influence of *M. tuberculosis* H37Rv (Mtb) hypoxic GRN on cellular processes

While evaluating the influence of hypoxic gene regulation on cellular processes, the set of proteins which are downstream to the transcription factors controlling hypoxia, and were ‘active’ (i.e. ‘turned-on’) during the hypoxic steady state (obtained through Boolean model simulations – see Supplementary Results), were considered for GO enrichment. The enriched GO terms thus obtained consisted of pathways like sulfate assimilation, metabolic compound salvage, cell cycle, etc. (Additional file 14). Amongst these, the sulfate assimilation pathway (GO:0000103) was observed to be around five fold up-regulated. This process has been reported to facilitate sulfate uptake from host and subsequently help in combating oxidative stress [37]. Interestingly, the iron-sulfur cluster assembly process (GO:0016226), was also obtained as one of the enriched (as well as up-regulated) pathways in the set of HPI associated Mtb proteins (as discussed in an earlier section). The results therefore indicate the role of iron-sulfur cluster assembly process and sulfate assimilation in conferring protection to Mtb against oxidative stress at late stages of infection.

The current analysis also showed around five fold up-regulation of nucleotide, nucleoside and purine containing compound salvage pathways (GO:0043174, GO:0043173 and GO:0043101 respectively) at late stage of infection. The role of pyrimidine salvage pathway in intra-cellular survival of Mtb at latent state has been implicated earlier [38]. Thus the observed enrichment of salvage pathways may pertain to the pathogen’s strategy for survival inside the host cell, while minimizing expenditure of cellular resources in de novo synthesis of the respective compounds.

In addition to the above mentioned pathways, cell cycle (GO:0007049) was also observed to be enriched, as well as up-regulated. Since this observation is contrary to the anticipated non-replicating physiology of Mtb inside the host cell, the exact roles of the genes/ proteins contributing to the observed enrichment were investigated. Out of six such proteins, three (EccC2, EccC3 and EccC5) were found to be homologs of a cell division protein FtsK. These proteins have been reported to belong to the ESAT-6 Secretion System (ESX), involved in virulence and viability of the pathogen [39, 40]. Wag31, one of the other proteins contributing to enrichment of cell cycle, was found to have a role in mitigation of oxidative stress [41] in addition to its role in cell division and cell shape regulation. Furthermore, the remaining two proteins from the list, viz. murC and murX, have been reported to be involved in peptidoglycan biosynthesis [42]. Therefore, the observed enrichment/ up-regulation of the ‘cell cycle’ process probably pertains to up-regulated pathogenic processes like ESX, mitigation of oxidative stress, cell shape regulation and peptidoglycan biosynthesis, and may not be indicative of cell replication.

Overall, the above observations suggest an up-regulation/ enrichment of cellular functions in Mtb which are helpful to counter the hostile environment of the host macrophage. This includes pathways for cell wall synthesis, sulfur assimilation, and oxidative stress mitigation. In addition, in an attempt to minimize its energy expenses, the Mtb cells probably utilize salvage pathways, especially for acquisition of nitrogen, iron and sulfur when present inside the macrophage.

#### Influence of HPI network and GRN on hypoxic metabolism of *M. tuberculosis* H37Rv (Mtb)

The previous sections pertained to understanding three different aspects of Mtb adaptation to the host intra-cellular environment. While the Mtb proteins involved in HPIs are expected to play a major role in sensing the host environment, its GRN is likely to govern the necessary adaptive changes. In other words, the shift in the metabolic paradigm during intra-cellular/ hypoxic adaptation can be expected to be driven by an altered flow of information through the host-pathogen interactome network and the gene regulatory cascade.

**Fig. 4:**
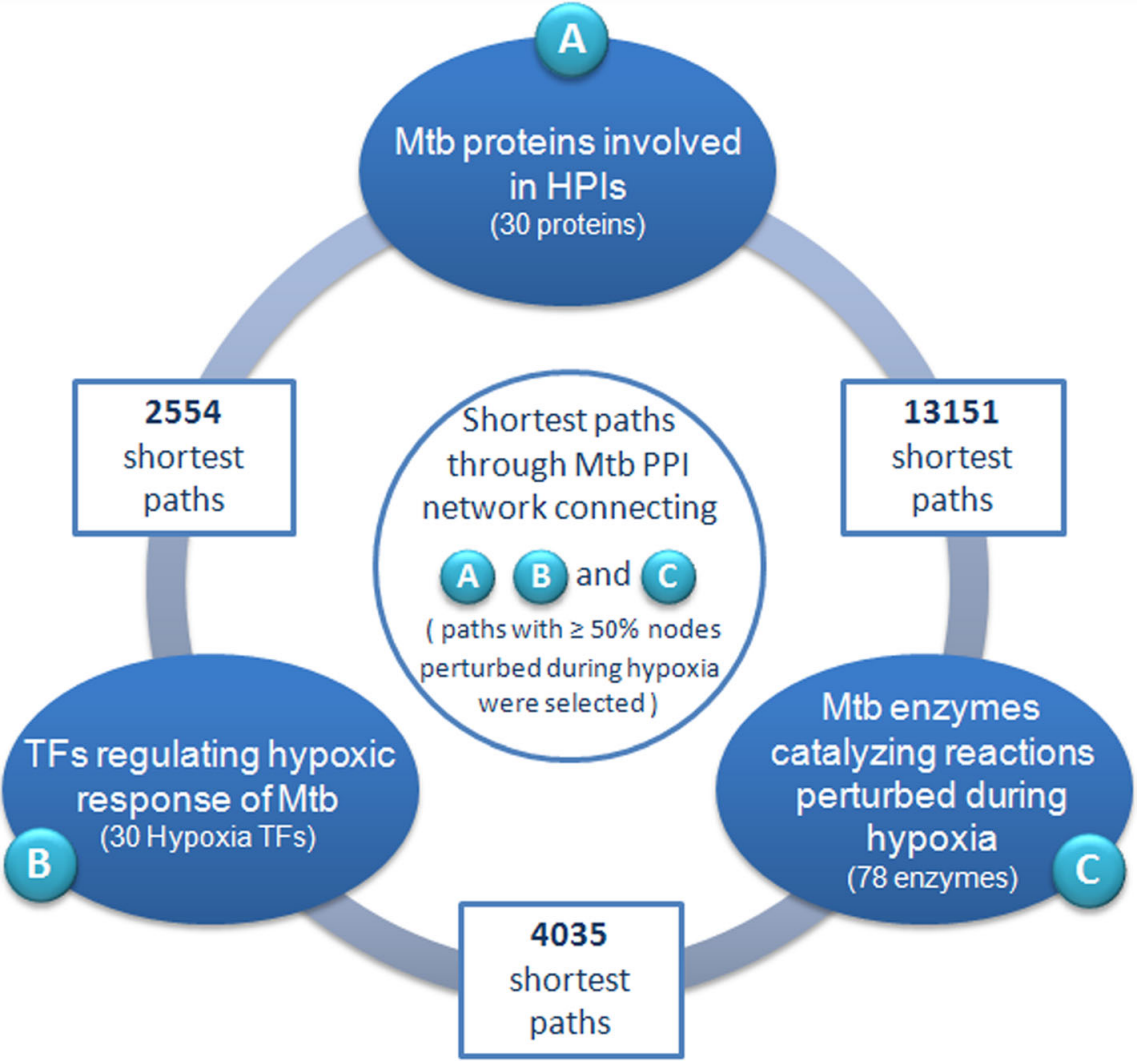
Number of identified shortest paths. Paths (of length ≤ 5) connecting (**a**) HPI-network, (**b**) the M. tuberculosis H37Rv (Mtb) hypoxic-GRN, and (**c**) the hypoxic-metabolism network of Mtb during its sustenance inside the host cell. The (shortest) paths were traced through a Mtb PPI network (derived from STRING database), and paths wherein at least 50% of the constituent nodes (genes/proteins) were observed to be perturbed during hypoxia were selected

In order to gauge the possible paths of information flow through and between (A) HPI-network, (B) the Mtb hypoxic-GRN, and (C) the hypoxic-metabolism network of Mtb during its sustenance inside the host cell, a ‘shortest-path analysis’ connecting individual components of the functional modules A, B and C was performed (details in Additional files 15). It was assumed that the shortest paths through the background PPI network would be the most optimal for carrying information from one functional module to the other. Information from the STRING database was utilized to build the background PPI network (details in Supplementary Methods). All shortest paths (of path-length < =5) between proteins involved in different modules were computed. Further, to ensure that the identified shortest paths had some relevance in flow of information between the studied modules, they were filtered based on gene expression of constituent nodes (during infection). Paths, wherein at-least 50% of the constituent genes were perturbed during hypoxia, were considered for further analysis (Additional file 3). The numbers of identified shortest paths between different modules are reported in Fig. 4. An integrated network of Mtb genes and proteins which could be involved in mediating information between (A) HPI-network, (B) the Mtb hypoxic-GRN, and (C) the hypoxic-metabolism network is presented in Additional files 16 and 17.

It was interesting to observe that a significant number of shortest paths (13151) connected the components of (A) HPI with (C) the enzymes identified to be the key modulators of metabolic flux during hypoxia. In contrast, the flow of information between (B) the TFs from hypoxic-GRN and (C) the metabolic enzymes, was mediated through 4035 shortest-paths. 2554 shortest-paths were observed between (A) the HPIs and (B) the hypoxic-GRN. One would expect that (B) the gene regulatory network controlling hypoxic response would have a significant effect on (C) the hypoxic metabolism. However, the above observations suggest that the effect of (A) the HPIs on (C) hypoxic metabolism may be far more pronounced. The findings are probably indicative of the fact that many TFs that could be responsible for adaptation of Mtb to hypoxia are not yet known or characterized. Consequently, the shortest paths between (A) HPI and (C) hypoxic-metabolism were screened to ascertain whether they consisted of any TFs which have not been reported to be associated with hypoxic gene regulation in any earlier study. Six Mtb TFs could be identified which are not part of (B) the hypoxic-GRN presented in this attempt. Two of these TFs, viz. Rv3676 and Rv3862c have been implicated in hypoxic response in recent studies [43, 44]. However, the exact regulatory cascades haven’t been identified yet. No literature evidence of association to hypoxia could be found for the remaining four identified TFs (Rv0097c, Rv1287, Rv1460 and Rv2359). However it was interesting to note that while Rv2359 has regulatory role in cell starvation (Bacon et al., 2014), Rv1460 is reported to be involved in mobilization of sulfur [36], both the processes being associated with intra-cellular survival of Mtb.

**Fig. 5:**
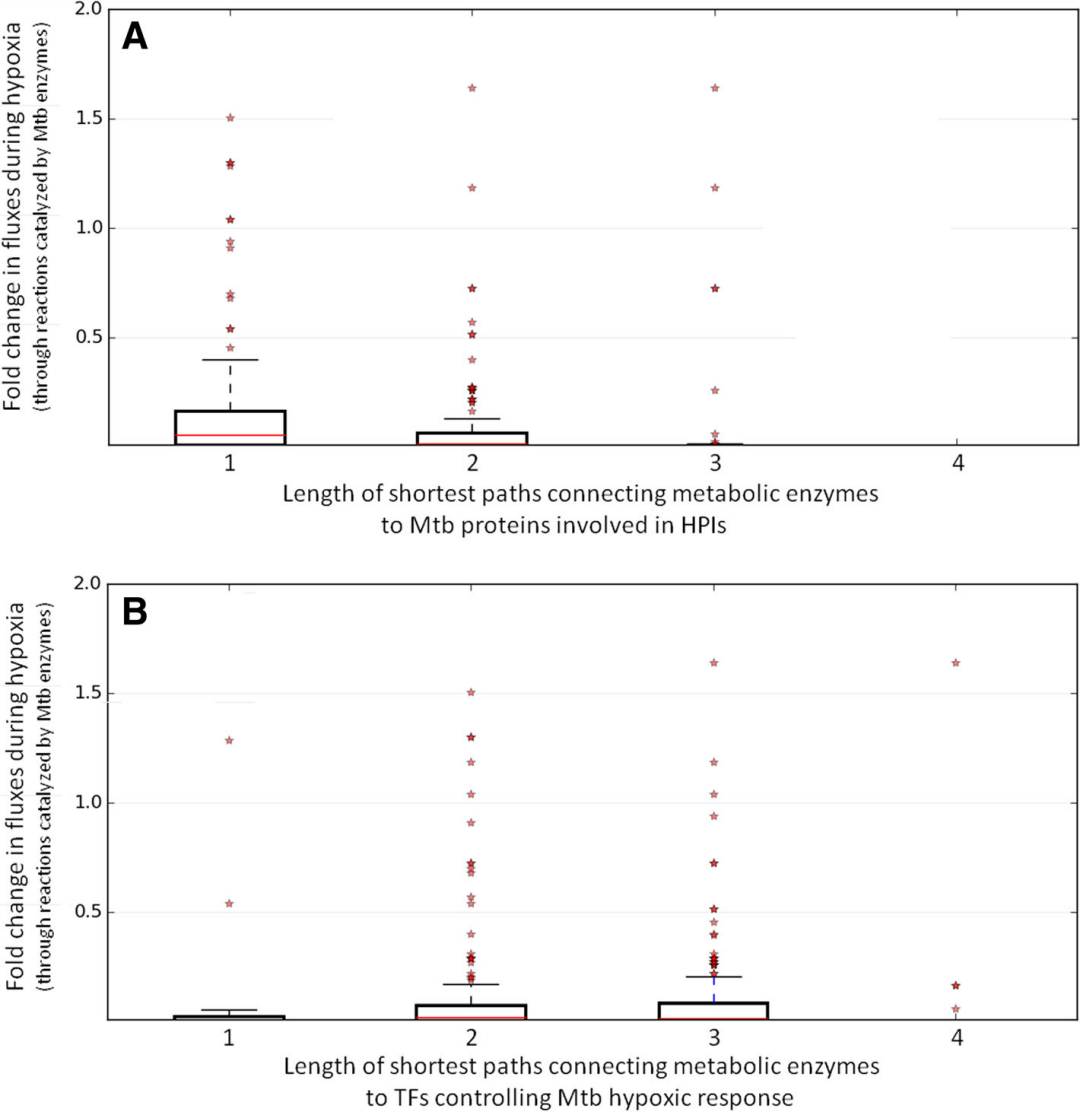
Plots depicting change in reaction fluxes during hypoxia versus lengths of shortest paths connecting metabolic enzymes to HPI and GRN networks. Plots depicting the magnitude of change in reaction fluxes during hypoxia (obtained using FBA simulations) versus the lengths of shortest paths (path lengths) connecting the corresponding enzymes to (**a**) M. tuberculosis H37Rv (Mtb) proteins involved in HPIs, and (**b**) transcription factors in the hypoxia gene regulatory network of Mtb. The median (red line), IQR (box), 1.5 × IQR (whisker), and outliers (red asterisks) corresponding to flux fold change values are indicated in the plot. The plot indicates that metabolic enzymes associated with reactions experiencing higher fold changes during hypoxia are more closely connected (in terms of shorter path lengths) to the proteins involved in HPIs, as compared to the transcription factors known to regulate hypoxic response

To further assess whether the connections (shortest paths) between (A) the HPI network and (C) metabolism were actually relevant in driving metabolic changes during hypoxia, the lengths of the paths connecting each of the metabolic enzymes (to the HPI network) with the fold-changes observed in the corresponding reaction fluxes during hypoxia were computed. The observations (Fig. 5) indicate that a shorter path-length corresponded to a higher fold change of flux, thereby emphasizing the probable ‘regulatory’ role of the HPI network on hypoxic metabolism.

## Discussion

In order to obtain a holistic view of the molecular changes that occur in *M. tuberculosis* H37Rv (Mtb) during the course of its adaptation to the host intra-cellular environment, we have analyzed various pertinent aspects using different computational approaches. These include – (i) the components (genes/proteins) that participate in interactions between the pathogen (Mtb) and the human (host), (ii) the Mtb gene regulatory network that may drive the adaptation of Mtb to hypoxia, and (iii) alterations in the metabolic pathways of Mtb in response to hypoxia.

Mtb as well as human components involved in the predicted set of HPIs were observed to be substantially connected, probably helping it to trigger a cascade of molecular events that are required for intra-cellular adaptation of Mtb once it senses the changes in environment. While some of these predicted HPIs provided important insights into probable pathogenic strategies (such as, HPI involving Mtb CipA-Human CDC42), others indicated at the host immune response (such as, HPI involving Mtb SodA-Human HLA) (also see Additional file 2). Further, the ‘KEGG Tuberculosis pathway (ko05152)’ was seen to encompass 89 (out of 148 human proteins) which were predicted to be involved in the HPIs. These findings re-emphasize the relevance of the predicted interactions and the proteins involved therein.

A key aspect of Mtb’s survival in the intra-cellular environment is its adaptation to hypoxia. Earlier studies dealing with this aspect of Mtb survival had identified several genes involved in the process through computational as well as experimental studies [7–9]. Given that most of these studies were disparate, an attempt was made to (a) collate all relevant information through literature mining, and (b) built a simulable model to study hypoxic gene regulation in Mtb. The genes regulated by *dosR*-induced initial response were seen to be enriched in functions like maintenance of cell wall, thereby indicating the pathogen’s preference to optimize resource or energy utilization for cell protection, before the onset of metabolic re-routing to utilize lipids and attain a viable but non-growing phenotype during the subsequent EHR stage. Further, simulation results identified a novel role for CsoR (Rv0967), which was observed to be a hub regulator of EHR. Given the known role of copper in mitigating oxidative stress (through SodC-mediation) in Mtb [45], further experiments investigating the regulatory effects of CsoR is likely to provide additional insights into Mtb virulence.

The hypoxic metabolism of Mtb was studied using FBA simulations. In contrast to earlier attempts [46], a minimal set of constraints (low oxygen uptake and a change in primary energy source) were employed to decipher the intrinsic pattern of flux re-routing during hypoxia (also see Additional file 10). Such changes are solely driven by the network architecture of the metabolic network and are expected to be the strongest determinant of metabolic changes during hypoxic adaptation. Mutant strains of Mtb lacking genes for glycolysis or gluconeogenesis and PPP were seen to be challenged for growth/ metabolism during hypoxia. These observations probably hint at a mechanism wherein Mtb counters the intra-cellular oxidative stress by generation of reducing equivalents through the PPP. Further, the scarcity of available sugars (coupled by arrested growth during infection) probably triggers Mtb to reverse the flow of flux through its glycolytic pathways to store glycogen for future use.

In addition to looking at the genes/ proteins/ enzymes directly involved in the investigated HPI, GRN or the metabolic network, the functional aspects of their neighbours were assessed in order to understand the influence of the former on other cellular processes. Despite no overlap between the constructed HPI network and the 24 TFs controlling hypoxic gene regulation, key cellular processes enriched in the ‘downstream’ PPI cascade of both these networks pertained to iron and sulfur assimilation and carbohydrate metabolism pathways involved in synthesis of cell wall components. Other processes associated with the HPI network which probably play an active role during intercellular survival of Mtb included PPP and synthesis of precursor metabolites. The hypoxic-GRN on the other hand had sulfate uptake, metabolic salvage pathways, and cell cycle amongst its list of downstream pathways/ processes that were seen to be modulated during hypoxia. While sulfate uptake and metabolic salvage pathways have obvious implication in a phagocytosed Mtb cell sourcing precursor metabolites from the host, redirection of flux towards cell wall synthesis and gluconeogenesis have earlier been reported in other studies [22, 47] as well as in our investigations into hypoxic metabolism. These hints indicating the possible overlap and inter connections between different cellular processes involved in Mtb’s intra-cellular survival led us to probe the links deeper through a shortest PPI path analysis. The results indicated a significantly enriched set of PPIs connecting the HPI network to the Mtb’s hypoxic metabolism. This was particularly intriguing, given that one would expect the GRN controlling hypoxic response to exert a more prominent effect on hypoxic metabolism. While our observations strengthen the premise of a concerted effort of different sensory and regulatory networks of Mtb in driving the metabolic changes during hypoxic adaptation, it raises a question regarding the completeness of our current understanding of TFs and gene regulation pertaining to hypoxia.

Experimental validation of certain interesting regulatory and metabolic aspects identified in this study can potentially enrich our current understanding of tuberculosis infection. Amongst the identified HPIs, proteins pertaining to two of the predicted interactions (‘Human Visfatin-Mtb GGT’ and ‘Human NINJ1-Mtb NarH’) with suspected role in subversion of host defence mechanisms, have been partially validated based on available literature and are potential candidates for further experimental validation. Experiments directed towards understanding the interaction dynamics of another predicted HPI, ‘Human p53-Mtb YchF’, would be intriguing given the critical role of p53 in cell cycle and virulence associated role of YchF. Further, validation of predicted interactions involving Mtb SodA and human immune system proteins could offer a new perspective on SodA-mediated mechanisms of immune modulation. With respect to the hypoxic gene regulatory network, the predicted crucial roles of the Mtb regulators CsoR and Rv0324 would be interesting aspects for experimental validation, which may aid in designing control strategies against latent tuberculosis. The *in silico* metabolic simulations in this work suggest that mutant strains of Mtb lacking genes for glycolysis or gluconeogenesis and PPP could be susceptible to impeded growth/ metabolism during hypoxia. Experimental validation of the above observation, by studying *Δfba*, *Δpgi*, and *ΔglpX* mutants in particular, can provide further understanding of Mtb metabolism during latency and stress.

## Conclusion

The role of HPIs and Mtb transcription factors in driving metabolic changes during hypoxic adaptation of *Mycobacterium tuberculosis* have been investigated in the current study. The insights obtained from the three connected aspects of intra-cellular survival of Mtb are expected to augment our current understanding of tuberculosis infection and guide in experimental design towards deeper investigations into host-pathogen interplay. In addition, the host-pathogen interaction network, the Boolean model of Mtb hypoxic gene-regulation, as well as the genome scale metabolic model of Mtb, presented in this study are expected to be useful resources for future studies on tuberculosis infection.

## Methods

A detailed methodology adopted for the various analysis performed in this study is provided in Additional files 1, 6, 10 and 15. In brief, the HPIs were predicted using an ‘interlogs’ based approach from a template PPI library as was reported in an earlier study [48]. The template PPI library was constructed from experimentally validated PPIs from various public databases (Additional file 1). In this method, for a given template PPI, A↔B (i.e., protein A interacting with protein B), the human/ Mtb orthologs of both A and B (A’ and B′ respectively) were first identified. Based on the inferred orthology, the following HPIs were inferred – (a) A ↔ B′ or (b) A’ ↔ B, where one of the interacting proteins belong to the host (human) and the other belong to the pathogen (refer to Figure S1.1). The predicted interactions were subsequently filtered (Additional file 1) based on several biologically relevant criteria (such as sub-cellular localization and expression levels of the interacting proteins) and finally collated into a host-pathogen interaction network.

A total of 286 genes/ proteins associated to hypoxia were mined from literature and subsequently categorized based on the time of gene expression into one of (i) *dosR* regulon genes, (ii) enduring hypoxic response (EHR) genes, and (iii) unclassified (genes lacking any time series expression data) as mentioned in Additional file 5. In addition, gene regulatory data (a total of 345 interactions) was retrieved through exhaustive literature search (Additional file 6). The hypoxia GRN comprising of 24 TFs (wherein each TF had interaction with at least one other TF) was then constructed and simulated using GINsim [49], a mathematical modelling tool using (multi-state) Boolean logics. Further, an extended GRN comprising of the ‘target’ genes (downstream genes) which are regulated by each of the 24 TFs in the above mentioned TF network were identified based on the TF-gene pairs collated from literature (Additional file 7). The extended GRN was also explored using a Boolean model in GINsim [49].

The available whole genome metabolic network of Mtb, iNJ661 (augmented with additional enzymes and reactions for lipid/ cholesterol metabolism) was used in the present study (details in Additional file 10). The augmented iNJ661 model (provided in Additional file 11) was simulated (using a COBRApy framework) for growth (with biomass as the objective function) using constraints mimicking M9 minimal media and varying carbon sources.

Shortest-paths connecting (A) the HPI-network, (B) the Mtb hypoxic-GRN, and (C) the hypoxic-metabolism network of Mtb were computed from the Mtb PPI network (as obtained from STRING database [50] and filtered for a combined score cut-off of 900), using the biological network analysis tool CompNet [51]. Further, paths having a maximal path-length of five and wherein, at least 50% of the genes were observed to be perturbed were considered as shortest-paths carrying information (details in Additional file 15). Subsequently, the statistical significance of the identified shortest-paths connecting any two modules (among A, B and C) was evaluated through t-tests.

## Additional files

Additional file 1: Details of the method used for prediction of host-pathogen interactions (HPIs) between human and *M. tuberculosis* H37Rv (Mtb) proteins. (DOCX 96 kb)

Additional file 2: Details of the Host-Pathogen Interactions (HPI) that were predicted between human and *M. tuberculosis* H37Rv (Mtb) cells. (DOCX 50 kb)

Additional file 3: Perturbations in (A) *M. tuberculosis* H37Rv (Mtb) and (B) human gene expression at different time-points post infection. The mean of expression values of a gene from different experimental datasets (collected from literature) has been considered. Expression values exhibiting at least 2-fold differential expression are indicated. (XLSX 2703 kb)

Additional file 4: Human pathways (KEGG biological pathways) associated with the host-pathogen interaction network. (DOCX 635 kb)

Additional file 5: List of genes involved in hypoxic response regulation of *M. tuberculosis* H37Rv. (XLSX 14 kb)

Additional file 6: Detailed method for analysis of gene regulatory network (GRN) controlling hypoxic response in *M. tuberculosis* H37Rv. (DOCX 412 kb)

Additional file 7: List of interacting “transcription factor - gene” pairs of *M. tuberculosis* H37Rv collated from literature. (XLSX 102 kb)

Additional file 8: Comparison of results of the multi-level Boolean model simulation with experimentally obtained gene expression data. (DOCX 71 kb)

Additional file 9: Results of the analysis on gene regulatory network (GRN) controlling hypoxic response in *M. tuberculosis* H37Rv. (DOCX 14 kb)

Additional file 10: Details of the adopted method and corresponding results of Flux Balance Analysis (FBA) of *M. tuberculosis* H37Rv metabolism during hypoxia. (DOCX 27 kb)

Additional file 11: The metabolic network model of *M. tuberculosis* H37Rv (Mtb) which was used for metabolic simulations in the current study (.xml format, can be used with MATLAB - COBRA toolbox or COBRApy framework; url- https://opencobra.github.io/). (XML 1043 kb)

Additional file 12: List of metabolic reactions in *M. tuberculosis* H37Rv which were significantly perturbed (over 2-fold) during hypoxia as compared to aerobic condition (results obtained through FBA simulations) (.xlsx format). (XLSX 14 kb)

Additional file 13: Significantly enriched GO biological process terms in the set of *M. tuberculosis* H37Rv proteins involved in HPIs are listed. Up-regulation or down-regulation of the biological processes during early and late infection time points, as inferred from the expression profiles of the genes constituting the respective processes, are also indicated. (XLSX 13 kb)

Additional file 14: Significantly enriched GO biological process terms in the set of *M. tuberculosis* H37Rv proteins, which were observed to be ‘active’/ switched ON in the ‘hypoxic’ stable state. This stable state was obtained through simulation of Boolean model corresponding to the gene regulatory network collated in the present study. (XLSX 10 kb)

Additional file 15: Details of the methodology adopted for identifying the shortest-paths among (A) the HPI-network, (B) the *M. tuberculosis* H37Rv hypoxic- gene regulatory network, and (C) the hypoxic-metabolism network of *M. tuberculosis* H37Rv. (DOCX 19 kb)

Additional file 16: *M. tuberculosis* H37Rv (Mtb) interaction network demonstrating the cross-talks between (A) HPI-network, (B) the Mtb hypoxic-GRN, and (C) the hypoxic-metabolism network during hypoxic adaptation. (XLSX 179 kb)

Additional File 17: *M. tuberculosis* H37Rv (Mtb) interaction network demonstrating the cross-talks between (A) HPI-network, (B) the Mtb hypoxic-GRN, and (C) the hypoxic-metabolism network during hypoxic adaptation (.cys format to be viewed using Cytoscape; url- http://www.cytoscape.org/). (CYS 313 kb)

## Abbreviations

EHR: Enduring hypoxic response
ESX: ESAT-6 secretion system
FBA: Flux balance analysis
GO: Gene ontology
GRN: Gene regulatory network
HPI: Host - pathogen interaction
Mtb: *Mycobacterium tuberculosis* H37Rv
PPI: Protein – protein interaction
PPP: Pentose phosphate shunt pathway
ROI/ RNI: Reactive oxygen intermediates/ Reactive nitrogen intermediates
TF: Transcription factor
