# Supplementary material for: Understanding the role of interactions between host and Mycobacterium tuberculosis under hypoxic condition: an in silico approach
Source: BMC Genomics. 2018 Jul 27;19:555. doi: 10.1186/s12864-018-4947-8 (PMC6064076; doi:10.1186/s12864-018-4947-8)
Supplement: Supplementary file 1 — Details of the method used for prediction of host-pathogen interactions (HPIs) between human and M. tuberculosis H37Rv (Mtb) proteins. (DOCX 96 kb) [file 12864_2018_4947_MOESM1_ESM.docx]

**Additional File 1: Details of the method used for prediction of host-pathogen interactions (HPIs) between human and *M. tuberculosis* H37Rv (Mtb) proteins**

The amount of experimentally validated data pertaining to host-pathogen (human-*M. tuberculosis*) interactions (HPIs) is limited [1]. A deeper insight into the virulence machinery of the pathogen necessitates adoption of computational methodologies towards identifying a broader set of HPIs [2]. Computational methods of HPI prediction are in general based on a template set of known protein-protein interactions (PPIs) (either intra-species PPIs or inter-species PPIs across different organisms), and make the assumption that function and interaction behaviour of orthologous proteins would be conserved/ similar [3]. When two proteins constituting a potential HPI pair bear significant homology (either at structural or at sequence level) with a corresponding pair of interacting proteins from the template PPI set, a host-pathogen interaction may be inferred [4,5]. Previous studies have used both sequence as well as structural homology based approaches for identifying new protein-protein interactions [1,4,4,6–9]. Given the limited number of available *M. tuberculosis* protein structures [2], a sequence homology based ‘interologs’ approach [7] was adopted in the present study with an aim of creating and utilizing a sufficiently large template PPI dataset. Furthermore, a refinement step was incorporated in our search, wherein each of the potential HPIs were validated considering contributing factors like cellular localization of the participating proteins, as well as their expression profiles during infection. The methodology for prediction of HPIs and construction of a protein-protein interaction network (PPI) has been elaborated below.

**Prediction of putative HPIs between human and *M. tuberculosis* H37Rv (Mtb) proteins**

A template PPI library was built after screening intra-species as well as inter-species PPIs from different publicly available (protein-protein interaction) databases. These databases include APID [10], BIND [11], DIP [12], HPIDB [13], I2D [14], InnateDB [15], Intact [16], MBinfo (www.mechanobio.info/), MINT [17], Molcon (www.molecularconnections.com/), MPIDB [18], PATRIC [19], and Spike [20]. Figure S1.1 provides a schematic representation of the homology/ interolog based method used to predict HPIs using the template library. For any given template PPI, A↔B (i.e., protein A interacting with protein B), the human/ Mtb orthologs of both A and B, say A' and B' respectively, were first identified. Orthology was inferred using reciprocal Blastp [21] using an e-value cut-off of 0.00001 and a coverage cut-off of 80%. While an identity cut-off of 30% was used for predicting homologues between humans and bacterial proteins, a cut-off of 50% was used between Mtb and other bacterial proteins. Subsequently, based on the inferred orthology, the following HPIs were inferred – (a) A ↔ B’ or (b) A’ ↔ B, provided one of the interacting proteins belongs to the host (human) and the other belongs to the pathogen (*M. tuberculosis* H37Rv).


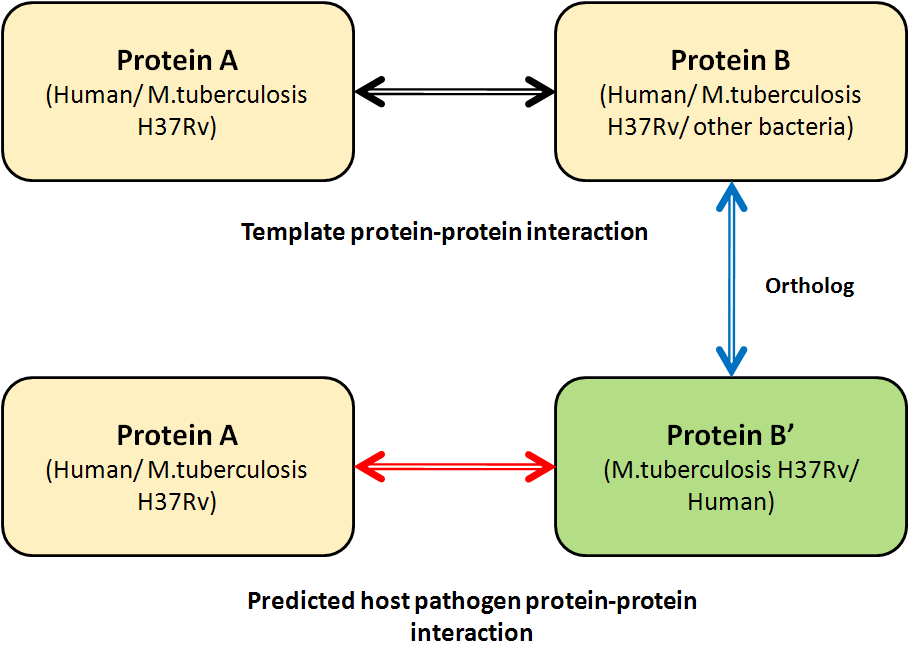


**Figure S1.1:** Schematic representation of the interolog based method used for predicting Host-Pathogen interactions (HPIs) between human and *M. tuberculosis* H37Rv proteins.

**Filtering of the predicted HPIs between human and *M. tuberculosis* H37Rv (Mtb) proteins**

The predicted set of HPIs was filtered based on a few relevant parameters. They included, (a) sub-cellular localisation, and (b) expression levels of interacting proteins during infection. It was assumed that (I) both the host and the pathogen proteins need to be co-localized, and (II) differentially expressed (as compared to basal expression levels) to take part in a HPI event. Proteins localized on the host and pathogen cell surfaces (and the secreted proteins) are the primary candidates for such HPIs. Further, in cases where the pathogen (Mtb) has been internalized by the host cell (e.g., engulfed by a macrophage), the pathogen proteins can also interact with the host’s cytoplasmic proteins. Sub-cellular localization of the human proteins was obtained from the Gene Ontology (GO) database [22]. Since, GO cellular component annotations were not available for most of the Mtb proteins, consensus of the results obtained from *in silico* approaches like PSORTb [23] and TBpred [24] was used for predicting the sub-cellular localizations of such Mtb proteins. Differentially expressed genes (during infection) for both the human and Mtb cells were extracted from available microarray datasets [25–27]. A gene was considered to be differentially expressed if it was two-fold up- /down-regulated (when compared to its basal expression level). A few experimentally validated HPIs [28–30] were also appended to the list of filtered HPIs to obtain the final list of predicted HPIs between human and Mtb cells.

**Construction of the PPI networks**

The HPIs obtained using the above mentioned interologs based prediction method and those mined from literature were used to construct the Mtb- human interactome HPI network. Further, high confidence intra-species PPIs from both human and Mtb were used to construct respective ‘background’ PPI networks. For this purpose, intra-species PPI networks (of both humans and Mtb) were separately downloaded from the STRING database [31]. The background PPI networks were further filtered for a cumulative score of greater than (or equal to) 900, as proposed by an earlier literature [32]. The topological properties of these networks were analyzed using Cytoscape [33].

**References**

1. Rapanoel HA, Mazandu GK, Mulder NJ. Predicting and analyzing interactions between Mycobacterium tuberculosis and its human host. PLoS ONE. 2013;8:e67472.

2. Zhou H, Jin J, Wong L. Progress in computational studies of host-pathogen interactions. J Bioinform Comput Biol. 2013;11:1230001.

3. Yu H, Luscombe NM, Lu HX, Zhu X, Xia Y, Han J-DJ, et al. Annotation transfer between genomes: protein-protein interologs and protein-DNA regulogs. Genome Res. 2004;14:1107–18.

4. Zhou H, Rezaei J, Hugo W, Gao S, Jin J, Fan M, et al. Stringent DDI-based prediction of H. sapiens-M. tuberculosis H37Rv protein-protein interactions. BMC Syst Biol. 2013;7 Suppl 6:S6.

5. Zhou H, Gao S, Nguyen NN, Fan M, Jin J, Liu B, et al. Stringent homology-based prediction of H. sapiens-M. tuberculosis H37Rv protein-protein interactions. Biol Direct. 2014;9:5.

6. Bose T, Venkatesh KV, Mande SS. Computational Analysis of Host-Pathogen Protein Interactions between Humans and Different Strains of Enterohemorrhagic Escherichia coli. Front Cell Infect Microbiol. 2017;7:128.

7. Dyer MD, Murali TM, Sobral BW. Computational prediction of host-pathogen protein-protein interactions. Bioinformatics. 2007;23:i159-166.

8. Mahajan G, Mande SC. Using structural knowledge in the protein data bank to inform the search for potential host-microbe protein interactions in sequence space: application to Mycobacterium tuberculosis. BMC Bioinformatics. 2017;18:201.

9. Raman K, Bhat AG, Chandra N. A systems perspective of host-pathogen interactions: predicting disease outcome in tuberculosis. Mol Biosyst. 2010;6:516–30.

10. Prieto C, De Las Rivas J. APID: Agile Protein Interaction DataAnalyzer. Nucleic Acids Res. 2006;34:W298-302.

11. Bader GD, Betel D, Hogue CWV. BIND: the Biomolecular Interaction Network Database. Nucleic Acids Res. 2003;31:248–50.

12. Xenarios I, Rice DW, Salwinski L, Baron MK, Marcotte EM, Eisenberg D. DIP: the database of interacting proteins. Nucleic Acids Res. 2000;28:289–91.

13. Kumar R, Nanduri B. HPIDB--a unified resource for host-pathogen interactions. BMC Bioinformatics. 2010;11 Suppl 6:S16.

14. Brown KR, Jurisica I. Online predicted human interaction database. Bioinformatics. 2005;21:2076–82.

15. Lynn DJ, Winsor GL, Chan C, Richard N, Laird MR, Barsky A, et al. InnateDB: facilitating systems-level analyses of the mammalian innate immune response. Mol Syst Biol. 2008;4:218.

16. Aranda B, Achuthan P, Alam-Faruque Y, Armean I, Bridge A, Derow C, et al. The IntAct molecular interaction database in 2010. Nucleic Acids Res. 2010;38:D525-531.

17. Ceol A, Chatr Aryamontri A, Licata L, Peluso D, Briganti L, Perfetto L, et al. MINT, the molecular interaction database: 2009 update. Nucleic Acids Res. 2010;38:D532-539.

18. Goll J, Rajagopala SV, Shiau SC, Wu H, Lamb BT, Uetz P. MPIDB: the microbial protein interaction database. Bioinformatics. 2008;24:1743–4.

19. Gillespie JJ, Wattam AR, Cammer SA, Gabbard JL, Shukla MP, Dalay O, et al. PATRIC: the comprehensive bacterial bioinformatics resource with a focus on human pathogenic species. Infect Immun. 2011;79:4286–98.

20. Paz A, Brownstein Z, Ber Y, Bialik S, David E, Sagir D, et al. SPIKE: a database of highly curated human signaling pathways. Nucleic Acids Res. 2011;39:D793-799.

21. Altschul SF, Gish W, Miller W, Myers EW, Lipman DJ. Basic local alignment search tool. J Mol Biol. 1990;215:403–10.

22. Ashburner M, Ball CA, Blake JA, Botstein D, Butler H, Cherry JM, et al. Gene ontology: tool for the unification of biology. The Gene Ontology Consortium. Nat Genet. 2000;25:25–9.

23. Gardy JL, Laird MR, Chen F, Rey S, Walsh CJ, Ester M, et al. PSORTb v.2.0: expanded prediction of bacterial protein subcellular localization and insights gained from comparative proteome analysis. Bioinformatics. 2005;21:617–23.

24. Rashid M, Saha S, Raghava GP. Support Vector Machine-based method for predicting subcellular localization of mycobacterial proteins using evolutionary information and motifs. BMC Bioinformatics. 2007;8:337.

25. Edgar R, Domrachev M, Lash AE. Gene Expression Omnibus: NCBI gene expression and hybridization array data repository. Nucleic Acids Res. 2002;30:207–10.

26. Karim AF, Chandra P, Chopra A, Siddiqui Z, Bhaskar A, Singh A, et al. Express path analysis identifies a tyrosine kinase Src-centric network regulating divergent host responses to Mycobacterium tuberculosis infection. J Biol Chem. 2011;286:40307–19.

27. Witney AA, Waldron DE, Brooks LA, Tyler RH, Withers M, Stoker NG, et al. BμG@Sbase—a microbial gene expression and comparative genomic database. Nucleic Acids Res. 2012;40:D605–9.

28. Cao W, Tang S, Yuan H, Wang H, Zhao X, Lu H. Mycobacterium tuberculosis antigen Wag31 induces expression of C-chemokine XCL2 in macrophages. Curr Microbiol. 2008;57:189–94.

29. Ghosh A, Tousif S, Bhattacharya D, Samuchiwal SK, Bhalla K, Tharad M, et al. Expression of the ARPC4 subunit of human Arp2/3 severely affects mycobacterium tuberculosis growth and suppresses immunogenic response in murine macrophages. PLoS ONE. 2013;8:e69949.

30. Tiwari B, Soory A, Raghunand TR. An immunomodulatory role for the Mycobacterium tuberculosis region of difference 1 locus proteins PE35 (Rv3872) and PPE68 (Rv3873). FEBS J. 2014;281:1556–70.

31. Franceschini A, Szklarczyk D, Frankild S, Kuhn M, Simonovic M, Roth A, et al. STRING v9.1: protein-protein interaction networks, with increased coverage and integration. Nucleic Acids Res. 2013;41:D808–15.

32. Lew JM, Kapopoulou A, Jones LM, Cole ST. TubercuList--10 years after. Tuberculosis (Edinb). 2011;91:1–7.

33. Shannon P, Markiel A, Ozier O, Baliga NS, Wang JT, Ramage D, et al. Cytoscape: a software environment for integrated models of biomolecular interaction networks. Genome Res. 2003;13:2498–504.
