# Supplementary material for: Understanding the role of interactions between host and Mycobacterium tuberculosis under hypoxic condition: an in silico approach
Source: BMC Genomics. 2018 Jul 27;19:555. doi: 10.1186/s12864-018-4947-8 (PMC6064076; doi:10.1186/s12864-018-4947-8)
Supplement: Supplementary file 2 — Details of the Host-Pathogen Interactions (HPI) that were predicted between human and M. tuberculosis H37Rv (Mtb) cells. (DOCX 50 kb) [file 12864_2018_4947_MOESM2_ESM.docx]

**Additional File 2: Details of the Host-Pathogen Interactions (HPI) that were predicted between human and *M. tuberculosis* H37Rv (Mtb) cells**

**Identification of host-pathogen interactions (HPIs) between human and *M. tuberculosis* H37Rv (Mtb)**

A comprehensive library of template PPIs, consisting of (a) 1,65,943 human-human intra-species PPIs, (b) 309 intra-species PPIs in different mycobacterial strains and (c) 8581 human-bacteria inter-species PPIs, was constructed using data retrieved from various public databases (see Methods). An interologs mapping approach (details in Additional File 1) using this template library could predict 4642 HPIs between 1853 human proteins and 590 *M. tuberculosis* H37Rv (Mtb) proteins. In order to evaluate the likelihood of occurrences of such interactions, it is also important to consider factors like cellular localization of the participating proteins, and/ or their expression profiles during infection. Since both the interacting proteins (host and pathogen proteins) need to be co-localized for successful interactions [1], the potential HPIs were filtered on the basis of the subcellular localizations of participating proteins (see Additional File 1). 381 HPIs consisting of 314 human proteins and 30 Mtb proteins were identified in this step. The current approach further considered perturbed expression levels of HPI genes (during infection) to be indicators of actual HPI events. Accordingly, the potential HPI list was further screened based on differential expression of both human and Mtb genes (details in Additional File 1). A list of 170 HPIs between 145 human and 26 Mtb proteins was thus obtained (Table S2.1). Although some HPIs involving constitutively expressed proteins (either in Mtb or the human cell) might be overlooked in this process, the predicted list is expected to have minimum (or no) incorrect (false positive) HPIs. Four experimentally elucidated HPIs [2–4] (Table S2.2) were further appended to this list to create the final human:Mtb interactome, constituting of 174 HPIs involving 148 human and 30 Mtb proteins (also see Figure 1).

**Table S2.1:** List of 174 identified HPIs between human and *M. tuberculosis* H37Rv (Mtb) proteins

| **Human-protein** | **Mtb-protein** |
| --- | --- |
| ACOX1 | MT1663 |
| AIMP2 | lysS2 |
| AIMP3 | lysS2 |
| AK5 | udgA |
| ALDH3A1 | MT0155 |
| AMY1B | sodB |
| AP1B1 | MT2098 |
| AP1G1 | narH |
| ARHGEF4 | lysS2 |
| ARPC4 | RVBD_1626 |
| ARRB2 | sdhA |
| AURKA | sodB |
| CA12 | atpG |
| CACNA1A | MT1663 |
| CASP6 | lysS2 |
| CASP8 | lysS2 |
| CAV1 | MT1663 |
| CD37 | lysS2 |
| CD53 | ggtB |
| CDC42 | Rv0479c |
| CDK4 | gnd2 |
| CDR1 | lepA |
| CNGB1 | ftsE |
| CREB3 | MT1663 |
| CUL1 | lysS2 |
| CUL4B | lysS2 |
| CYHR1 | MT0155 |
| CYP4F12 | sodB |
| DYNC1LI1 | sodB |
| DYSF | lysS2 |
| E2F1 | MT1510 |
| EEF2 | lysS2 |
| EGFR | atpG |
| EGR2 | nuoD |
| EIF1B | sodB |
| EIF2A | lysS2 |
| EMG1 | lysS2 |
| EPB41 | MT1663 |
| EPRS | lysS2 |
| FN1 | MT1143 |
| FN1 | lysS2 |
| FOXO3 | sodB |
| FXN | sdhA |
| FXN | sdhB |
| **Human-protein** | **Mtb-protein** |
| GABARAPL1 | sdhA |
| GABARAPL1 | echA21 |
| GABARAPL1 | sdhB |
| GABARAPL1 | lysS2 |
| GADD45G | MT0074 |
| GAPDH | lysS2 |
| GBE1 | gnd2 |
| GDPD2 | acn |
| GEM | sdhB |
| GEMIN4 | lysS2 |
| GOLGB1 | sodB |
| GPRC5C | MT1143 |
| HDHD2 | sodB |
| HIST2H2BE | lysS2 |
| HLA | sodB |
| HLA | sdhA |
| HLA | sdhB |
| HLA | gnd2 |
| HNF4A | MT3321 |
| HNF4A | lepA |
| HNF4A | MT0155 |
| HNF4A | oxcA |
| HNF4A | lysS2 |
| HNF4A | udgA |
| HNF4A | MT1510 |
| HNF4A | MT0966 |
| HSPE1 | lepA |
| IARS | lysS2 |
| ICT1 | accD4 |
| ICT1 | nuoD |
| ICT1 | echA21 |
| ICT1 | lysS2 |
| IFIT1 | accD4 |
| IGFBP3 | pgi |
| ITIH2 | atpG |
| JUN | pgi |
| KARS | lysS2 |
| KCNJ11 | MT2098 |
| KIAA1549 | sodB |
| LARS | lysS2 |
| LGALS3 | MT2098 |
| MAPK6 | oxcA |
| MARS | lysS2 |
| MBNL1 | MT2098 |
| MCC | sodB |
| MCC | echA21 |
| MCC | sdhB |
| MLF1IP | nuoD |
| MPG | sodB |
| **Human-protein** | **Mtb-protein** |
| MYH8 | sodB |
| NAMPT | ggtB |
| NDUFA9 | nuoD |
| NDUFS8 | nuoD |
| NGEF | atpG |
| NINJ1 | narH |
| P4HB | sodB |
| PAFAH1B3 | echA21 |
| PAWR | atpG |
| PCCB | accD4 |
| PCNA | lysS2 |
| PEX5 | echA21 |
| PHB | nuoD |
| PIK3R3 | lysS2 |
| PIK3R5 | acn |
| PIK3R5 | sdhB |
| PITPNA | MT1663 |
| POLA2 | atpG |
| PPP2R2B | accD4 |
| PPP2R2B | echA21 |
| PRPF3 | lysS2 |
| PTN | atpG |
| RAD51 | udgA |
| RIPK2 | sodB |
| RPL11 | lysS2 |
| RPL14 | lysS2 |
| RPL18 | lysS2 |
| RPL21 | sodB |
| RPL21 | lysS2 |
| RPL24 | lysS2 |
| RPL27 | lysS2 |
| RPL30 | lysS2 |
| RPL35A | lysS2 |
| RPL5 | lysS2 |
| RPL6 | lysS2 |
| RPL7 | lysS2 |
| RPL8 | lysS2 |
| RPL9 | lysS2 |
| RPS11 | lysS2 |
| RPS13 | lysS2 |
| RPS2 | lysS2 |
| RPS26 | lysS2 |
| RPS3 | lysS2 |
| RPS3A | sodB |
| RPS4X | lysS2 |
| RPS6 | lysS2 |
| RPS8 | lysS2 |
| SCRN1 | sodB |
| SDS | sdhB |
| **Human-protein** | **Mtb-protein** |
| SERPINB9 | echA21 |
| SFRP4 | MT1143 |
| SGK1 | MT1143 |
| SIRT3 | udgA |
| SLC25A5 | MT0074 |
| SLC25A6 | lysS2 |
| SOD2 | sodB |
| SPRY2 | sdhB |
| SQSTM1 | nuoD |
| SRSF3 | lepA |
| SSRP1 | lysS2 |
| STAT3 | echA21 |
| TANK | lysS2 |
| TIMP1 | echA21 |
| TLR10 | echA21 |
| TLR2 | PE35 |
| TLR2 | PPE68 |
| TNFRSF14 | nuoD |
| TNIK | sodB |
| TP53 | MT1143 |
| TRAF6 | gnd2 |
| TRIP10 | MT1143 |
| TUT1 | echA21 |
| TUT1 | lysS2 |
| USPL1 | gnd2 |
| VCAM1 | sdhA |
| VCAM1 | sdhB |
| VHL | sodB |
| WBP5 | sodB |
| XCL2 | RVBD_2145c |
| YWHAG | lysS2 |
| ZMYND19 | oxcA |

**Table S2.2:** List of the experimentally validated HPIs involving human and *M. tuberculosis* H37Rv (Mtb) proteins (which were included in the current study)

| **Human-protein** | **Mtb-protein** | **Reference** |
| --- | --- | --- |
| TLR2 | PE35 | doi:10.1111/febs.12723 |
| TLR2 | PPE68 | doi:10.1111/febs.12723 |
| ARPC4 | RVBD_1626 | doi:10.1371/journal.pone.0069949 |
| XCL2 | RVBD_2145c | doi:10.1007/s00284-008-9172-2 |

**Insights from the human and *M. tuberculosis* H37Rv (Mtb) interactome network**

A thorough literature curation provided evidences in support of several of the identified HPIs and their probable role in pathogenesis and/or host immune responses. For instance, an interaction between the human protein CDC42 and Mtb protein CipA (Rv0479c) has been predicted. Both *cipA* and *CDC42* were found to be up-regulated (from the microarray gene expression profiles – Additional File 3) when the host macrophages (or monocyte derived macrophages, THP1 cell line) were infected with Mtb. CDC42 activates cytoskeletal rearrangement in macrophages and has been reported to be controlled by the CipA protein in *Mycobacterium avium* infection model [5]. The association of cytoskeletal rearrangements of the host cell with the phagocytic uptake of invading pathogens has also been reported in earlier studies [6,7]. It may further be noted that tuberculosis-specific databases like Tuberculist [8] and targetTB [9] have reported *cipA* to be an essential gene for growth, as well as a potential drug target. The above observations hint at the probable role of CipA in modulating the bacterial load (inside host cells) by influencing cytoskeletal rearrangement (of host cells).

Another predicted HPI involved the human nicotinamide phosphoribosyltransferase (Visfatin) and Mtb gamma-glutamyltransferase (GGT/Rv2394) proteins. While the gene expression datasets suggested up-regulation of human visfatin gene under infection condition, the mycobacterial *ggt* was observed to be down-regulated (Additional File 3). Earlier studies have reported that higher concentration of visfatin increases cellular levels of glutathione (GSH) by inhibiting glutathione peroxidase (GSH-Px) [10]. GSH is an antioxidant and is found in host phagocytic cells (like macrophage). GSH helps in protecting the host cell from the reactive oxygen intermediates (ROI) and reactive nitrogen intermediates (RNI), which are generated in response to pathogenic infection [11]. Further, nitric oxide (NO) is known to react with GSH to form S-nitrosoglutathione (GSNO), a highly potent bactericidal agent. A previous study has shown that GSNO is cleaved by mycobacterial GGT into a dipeptide [Cys(NO)-Gly] and glutamate, followed by the entry of Cys(NO)-Gly into the bacterium aided by the activity of dipeptide permease [11]. Thus the predicted HPI (involving Visfatin and GGT), viewed in context of the above facts, leads us to hypothesize that the down-regulation of GGT in Mtb is probably a response to increased visfatin/GSH levels in the macrophage. This might be one of the defensive strategies adopted by the pathogen for evading NO mediated stress.

In yet another identified HPI, the human protein, ninjurin (NINJ1) was seen to interact with nitrate reductase (NarH) of Mtb. Gene expression profiles (Additional File 3) indicated *ninj1* to be up-regulated during infection (data available at 4-hours post infection). The *narH* gene was observed to be up-regulated till 18 hours post infection and down-regulated thereafter. Ninjurin has previously been reported to be up-regulated on induction of inflammation, and in turn, shown to induce iNOS (inducable Nitric Oxide Synthase) expression as well as increased NO generation [12]. Subsequently, NO (under oxidative stress conditions) interacts with superoxide to form peroxynitrite, which is an unstable isomer of nitrate [13]. Therefore, the predicted HPI involving NINJ1 and NarH assumes importance, considering the probable role of mycobacterial nitrate reductase (NarH) in reducing this nitrate mediated stress.

The Mtb SodA protein, also known to be involved in oxidative stress response, was predicted to be involved in interactions with several human proteins. These human proteins included cytochrome P450, cytoplasmic dynein, forkhead box protein O3, myosin, receptor-interacting serine/threonine-protein kinase 2, Secernin-1, TRAF2, HLA class I, NCK-interacting protein kinase, etc. The above observations re-emphasize a key role of SodA in mycobacterial virulence. The potential HPI between human HLA class I histocompatibility antigen and the Mtb SodA adds another perspective pertaining to host immune adaptations towards subverting the pathogen. This interaction probably pertains to the innate immune mechanism of the host, with HLA aiding in presenting Mtb SodA fragments on the host cell surface. It is pertinent to note in this context that an earlier study [14] had reported the presence of a HLA specific epitopic region within the mycobacterial SodA. Given that SodA (involved in mitigating oxidative stress) is expected to be highly expressed in Mtb cells internalized by the phagosomes, the predicted interaction of human HLA with Mtb SodA may facilitate efficient antigen presentation.

Another interesting predicted HPI pertained to the human tumor suppressor protein (p53) and the ribosome-binding ATPase (YchF) of Mtb. This is particularly significant given the role of p53 in modulation of apoptosis. YchF, on the other hand, has previously been reported to play a key role in macrophage cytotoxicity, iron acquisition and virulence in studies pertaining to *Vibrio vulnifucus* infected mouse models [15]. An earlier study [16] of human-Mtb HPIs had also predicted a number of interactions connected to the cancer related pathways. Although these observations may be attributed to the significant overlap between cancer pathways and infection-related processes [16], detailed investigation is required to understand the role of these HPIs in modulating the cell-cycle and associated human pathways.

**References**

1. Yellaboina S, Goyal K, Mande SC. Inferring genome-wide functional linkages in E. coli by combining improved genome context methods: comparison with high-throughput experimental data. Genome Res. 2007;17:527–35.

2. Cao W, Tang S, Yuan H, Wang H, Zhao X, Lu H. Mycobacterium tuberculosis antigen Wag31 induces expression of C-chemokine XCL2 in macrophages. Curr Microbiol. 2008;57:189–94.

3. Ghosh A, Tousif S, Bhattacharya D, Samuchiwal SK, Bhalla K, Tharad M, et al. Expression of the ARPC4 subunit of human Arp2/3 severely affects mycobacterium tuberculosis growth and suppresses immunogenic response in murine macrophages. PLoS ONE. 2013;8:e69949.

4. Tiwari B, Soory A, Raghunand TR. An immunomodulatory role for the Mycobacterium tuberculosis region of difference 1 locus proteins PE35 (Rv3872) and PPE68 (Rv3873). FEBS J. 2014;281:1556–70.

5. Harriff MJ, Danelishvili L, Wu M, Wilder C, McNamara M, Kent ML, et al. Mycobacterium avium genes MAV_5138 and MAV_3679 are transcriptional regulators that play a role in invasion of epithelial cells, in part by their regulation of CipA, a putative surface protein interacting with host cell signaling pathways. J Bacteriol. 2009;191:1132–42.

6. Bermudez LE, Goodman J. Mycobacterium tuberculosis invades and replicates within type II alveolar cells. Infect Immun. 1996;64:1400–6.

7. García-Pérez BE, De la Cruz-López JJ, Castañeda-Sánchez JI, Muñóz-Duarte AR, Hernández-Pérez AD, Villegas-Castrejón H, et al. Macropinocytosis is responsible for the uptake of pathogenic and non-pathogenic mycobacteria by B lymphocytes (Raji cells). BMC Microbiol. 2012;12:246.

8. Lew JM, Kapopoulou A, Jones LM, Cole ST. TubercuList--10 years after. Tuberculosis (Edinb). 2011;91:1–7.

9. Raman K, Yeturu K, Chandra N. targetTB: a target identification pipeline for Mycobacterium tuberculosis through an interactome, reactome and genome-scale structural analysis. BMC Syst Biol. 2008;2:109.

10. Bułdak RJ, Bułdak Ł, Polaniak R, Kukla M, Birkner E, Kubina R, et al. Visfatin affects redox adaptative responses and proliferation in Me45 human malignant melanoma cells: an in vitro study. Oncol Rep. 2013;29:771–8.

11. Dayaram YK, Talaue MT, Connell ND, Venketaraman V. Characterization of a glutathione metabolic mutant of Mycobacterium tuberculosis and its resistance to glutathione and nitrosoglutathione. J Bacteriol. 2006;188:1364–72.

12. Kim K-W, Lee H-J. Composition comprising expression or activity inhibitors of ninjurin 1 for the prevention and treatment of inflammatory disease [Internet]. 2011 [cited 2017 May 12]. Available from: http://www.google.com/patents/US20110123538

13. Ischiropoulos H, Zhu L, Chen J, Tsai M, Martin JC, Smith CD, et al. Peroxynitrite-mediated tyrosine nitration catalyzed by superoxide dismutase. Arch Biochem Biophys. 1992;298:431–7.

14. Dong Y, Demaria S, Sun X, Santori FR, Jesdale BM, De Groot AS, et al. HLA-A2-restricted CD8+-cytotoxic-T-cell responses to novel epitopes in Mycobacterium tuberculosis superoxide dismutase, alanine dehydrogenase, and glutamine synthetase. Infect Immun. 2004;72:2412–5.

15. Chen Y-C, Chung Y-T. A conserved GTPase YchF of Vibrio vulnificus is involved in macrophage cytotoxicity, iron acquisition, and mouse virulence. Int J Med Microbiol. 2011;301:469–74.

16. Zhou H, Gao S, Nguyen NN, Fan M, Jin J, Liu B, et al. Stringent homology-based prediction of H. sapiens-M. tuberculosis H37Rv protein-protein interactions. Biol Direct. 2014;9:5.
