# Supplementary material for: Understanding the role of interactions between host and Mycobacterium tuberculosis under hypoxic condition: an in silico approach
Source: BMC Genomics. 2018 Jul 27;19:555. doi: 10.1186/s12864-018-4947-8 (PMC6064076; doi:10.1186/s12864-018-4947-8)
Supplement: Supplementary file 4 — Human pathways (KEGG biological pathways) associated with the host-pathogen interaction network. (DOCX 635 kb) [file 12864_2018_4947_MOESM4_ESM.docx]

**Additional File 4: Human pathways associated with the host-pathogen interaction network**

In order to gain deeper insights into how human pathways may be influenced during Mtb infection, the association of the human proteins involved in the identified HPIs to different KEGG pathways [1] were investigated. Table S4.1 lists the human KEGG pathways, which included host proteins associated with the identified HPIs. Pathways which consisted of 5 or more HPI associated proteins are listed. The KEGG Tuberculosis pathway (ko05152) topped the list, wherein as many as 89 proteins (out of a total of 148 human proteins involved in HPIs) were found to be associated (Figure S4.1). This observation is aligned to our expectations since the KEGG Tuberculosis pathway lists the key biological processes (and the constituent human proteins) that are known to be affected by Mtb infection. The HPI proteins (associated to KEGG Tuberculosis pathway) identified in the present analysis is a significant addition to the previously reported smaller set [2]. Human proteins (from the predicted HPI network), pertaining to host mechanisms like apoptosis, Toll-like receptor signalling, NOD-like receptor signalling, MAPK signalling, production of pro-inflamatory cytokines and Type-I inteferons, were observed to be connected to the Mtb mediated virulence factors included in the KEGG Tuberculosis pathway. 13 host proteins (involved in HPIs) were also found to be part of the PI3K-Atk signalling pathway. Given the role of PI3K-Atk signalling in controlling cell cycle and apoptosis [3], this observation assumes significance. A deeper look into the up-/down-regulation patterns of these 13 proteins (Additional File 3) indicated a G1/S phase transition of the infected cells [4]. Similar observations have been attributed to the long term residence of the pathogen *Mycobacterium leprae* inside the host cells [5].

**Table S4.1:** List of the (KEGG) biological pathways which includes multiple (at least 5) host proteins participating in HPIs with *M. tuberculosis* H37Rv (Mtb). The pathways are sorted in descending order of their HPI-associated protein content.

| **Pathway-ID** | **Genes mapped** | **Pathway** |
| --- | --- | --- |
| ko05152 | 89 | Tuberculosis - Homo sapiens (human) |
| hsa03010 | 21 | Ribosome |
| hsa01100 | 14 | Metabolic pathways |
| hsa05200 | 14 | Pathways in cancer |
| hsa05203 | 13 | Viral carcinogenesis |
| hsa04151 | 13 | PI3K-Akt signaling pathway |
| hsa05166 | 12 | HTLV-I infection |
| hsa05161 | 12 | Hepatitis B |
| hsa05205 | 10 | Proteoglycans in cancer |
| hsa05168 | 10 | Herpes simplex infection |
| hsa05212 | 9 | Pancreatic cancer |
| hsa04068 | 9 | FoxO signaling pathway |
| hsa04010 | 8 | MAPK signaling pathway |
| hsa05016 | 8 | Huntington's disease |
| hsa04066 | 8 | HIF-1 signaling pathway |
| hsa05169 | 8 | Epstein-Barr virus infection |
| hsa05160 | 8 | Hepatitis C |
| hsa04722 | 8 | Neurotrophin signaling pathway |
| hsa04810 | 7 | Regulation of actin cytoskeleton |
| hsa05223 | 7 | Non-small cell lung cancer |
| hsa04152 | 7 | AMPK signaling pathway |
| hsa04110 | 7 | Cell cycle |
| hsa04062 | 7 | Chemokine signaling pathway |
| hsa05162 | 7 | Measles |
| hsa05222 | 7 | Small cell lung cancer |
| hsa04510 | 7 | Focal adhesion |
| hsa04932 | 7 | Non-alcoholic fatty liver disease (NAFLD) |
| hsa05142 | 7 | Chagas disease (American trypanosomiasis) |
| hsa04022 | 6 | cGMP-PKG signaling pathway |
| hsa04668 | 6 | TNF signaling pathway |
| hsa05218 | 6 | Melanoma |
| hsa04144 | 6 | Endocytosis |
| hsa05145 | 6 | Toxoplasmosis |
| hsa05214 | 6 | Glioma |
| hsa04024 | 6 | cAMP signaling pathway |
| hsa05100 | 6 | Bacterial invasion of epithelial cells |
| hsa05215 | 6 | Prostate cancer |
| hsa04620 | 6 | Toll-like receptor signaling pathway |
| hsa04921 | 5 | Oxytocin signaling pathway |
| hsa04380 | 5 | Osteoclast differentiation |
| hsa05206 | 5 | MicroRNAs in cancer |
| hsa04915 | 5 | Estrogen signaling pathway |
| hsa05211 | 5 | Renal cell carcinoma |
| hsa04210 | 5 | Apoptosis |
| hsa05213 | 5 | Endometrial cancer |
| hsa04530 | 5 | Tight junction |
| hsa04660 | 5 | T cell receptor signaling pathway |
| hsa05220 | 5 | Chronic myeloid leukemia |
| hsa00970 | 5 | Aminoacyl-tRNA biosynthesis |
| hsa05146 | 5 | Amoebiasis |
| hsa04115 | 5 | p53 signaling pathway |

**
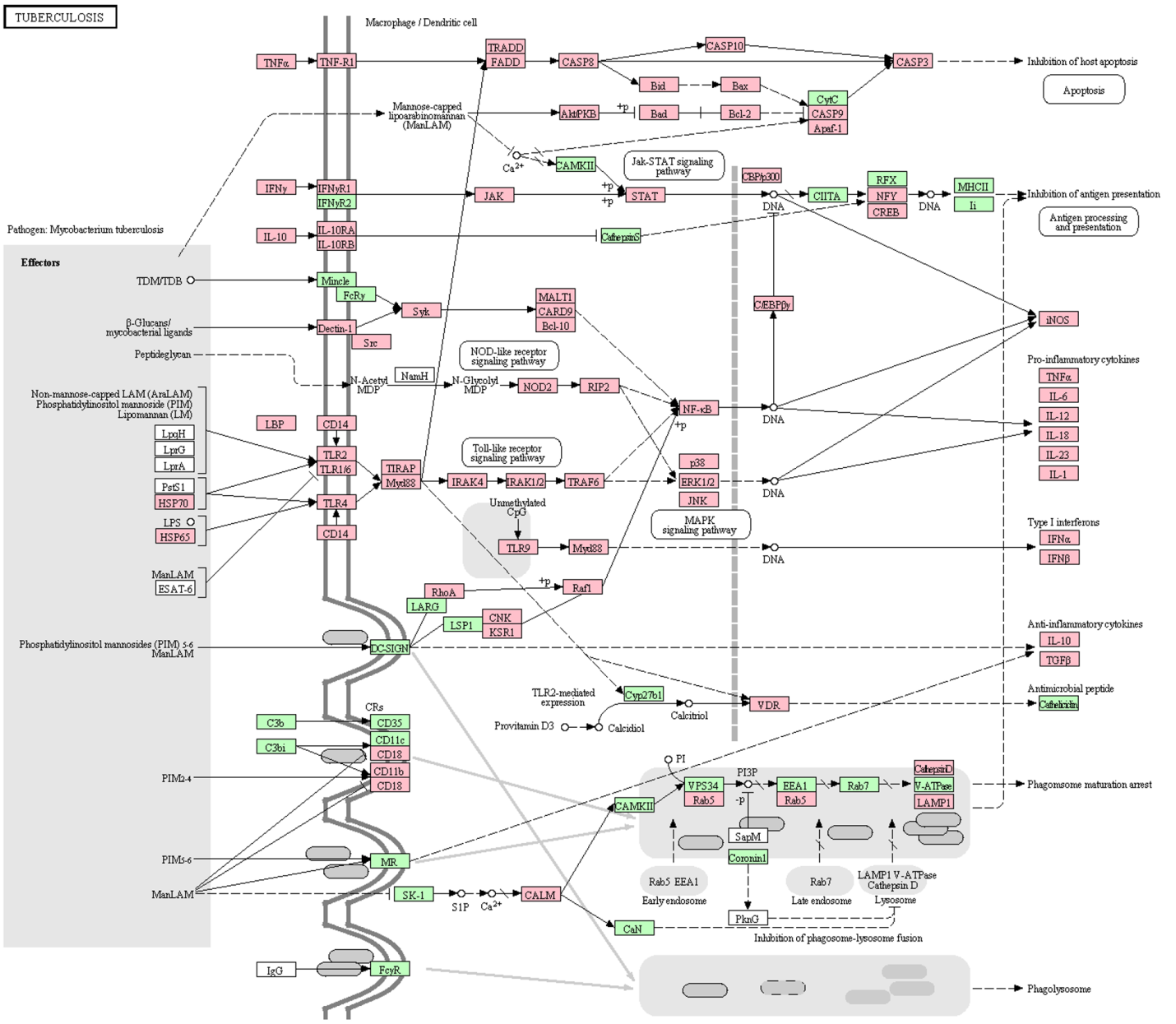
**

Figure S4.1: KEGG Tuberculosis pathway highlighting (in pink) the human proteins involved in HPIs identified in this study.

**References**

1. Kanehisa M, Furumichi M, Tanabe M, Sato Y, Morishima K. KEGG: new perspectives on genomes, pathways, diseases and drugs. Nucleic Acids Res. 2017;45:D353–61.

2. Rapanoel HA, Mazandu GK, Mulder NJ. Predicting and analyzing interactions between Mycobacterium tuberculosis and its human host. PLoS ONE. 2013;8:e67472.

3. Chang F, Lee JT, Navolanic PM, Steelman LS, Shelton JG, Blalock WL, et al. Involvement of PI3K/Akt pathway in cell cycle progression, apoptosis, and neoplastic transformation: a target for cancer chemotherapy. Leukemia. 2003;17:590–603.

4. Vadlakonda L, Pasupuleti M, Pallu R. Role of PI3K-AKT-mTOR and Wnt Signaling Pathways in Transition of G1-S Phase of Cell Cycle in Cancer Cells. Front Oncol. 2013;3:85.

5. Tapinos N, Rambukkana A. Insights into regulation of human Schwann cell proliferation by Erk1/2 via a MEK-independent and p56Lck-dependent pathway from leprosy bacilli. Proc Natl Acad Sci USA. 2005;102:9188–93.
