# Supplementary material for: Understanding the role of interactions between host and Mycobacterium tuberculosis under hypoxic condition: an in silico approach
Source: BMC Genomics. 2018 Jul 27;19:555. doi: 10.1186/s12864-018-4947-8 (PMC6064076; doi:10.1186/s12864-018-4947-8)
Supplement: Supplementary file 6 — Detailed method for analysis of gene regulatory network (GRN) controlling hypoxic response in M. tuberculosis H37Rv. (DOCX 412 kb) [file 12864_2018_4947_MOESM6_ESM.docx]

**Additional File 6: Detailed method for analysis of gene regulatory network (GRN) controlling hypoxic response in *M. tuberculosis* H37Rv (Mtb)**

**Collation of *M. tuberculosis* H37Rv (Mtb) genes involved in hypoxic response**

Literature was mined to identify all genes previously reported to be associated to hypoxic response of Mtb [1–8]. 286 genes found to be associated to the hypoxic response were classified under three categories – (i) those involved in initial hypoxic response or *dosR* regulon genes, (ii) those implicated in enduring hypoxic response (EHR), i.e. EHR genes and (iii) those which are unclassified (genes lacking any time series expression data) based on the time of gene expression (immediately after infection or later) (Additional File 5). These categories consisted of 57, 230 and 4 genes respectively (with 5 overlapping genes in the *dosR* regulon and EHR). Analysis of the associated biological pathways for these sets of genes was performed using Gene ontology (GO) cellular component terms [9]. The procedure adopted for performing the GO enrichment analysis has been detailed in a subsequent section.

**Construction of transcription factor network (TF network) involved in *M. tuberculosis* H37Rv (Mtb) hypoxic response**

Gene regulatory data pertaining to the adaptation of Mtb to hypoxic environment was obtained through exhaustive literature search. Apart from studies directed towards functional characterization of one or a few genes of interest, high-throughput experimental studies were also considered while collating the gene regulatory information. A total of 345 interactions could be retrieved, where the transcription factors (TFs) have been reported to significantly induce or reduce the expression level of the target gene under hypoxic environment [3,7,10–15]. Subsequently, a TF network was constructed where each TF had interaction with at least one other TF. This network was observed to contain 24 nodes (TFs) and 71 edges. Additionally, in order to model the effects of external regulators and uncharacterized internal regulators on the expression of TFs, one node representing ‘external or internal regulators’ was incorporated in the TF network. Consequently, two additional edges were incorporated in the network connecting ‘external/internal regulators’ to the two input nodes (Rv0767c and Rv2324) having only outgoing interactions. Also, a self inducing edge was attached to the node ‘external/internal regulators’ to provide a time dependent input stimulus to the network. The TF network (Figure S6.1) thus obtained from the collated regulatory interactions was further analyzed through mathematical modelling.


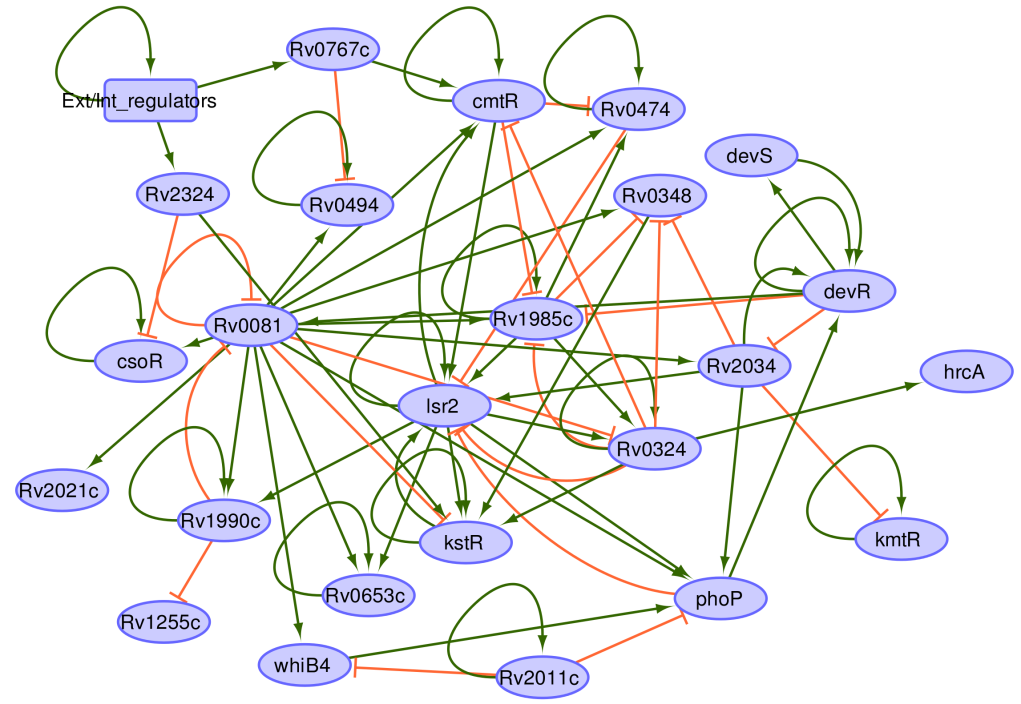


Figure S6.1: Schematic representation of the transcription factor network (TF network) controlling hypoxic response in *M. tuberculosis* H37Rv (Mtb). The green and red coloured edges represent positive and negative regulations, respectively.

**Construction and simulation of the multi-state Boolean model mimicking hypoxic TF network**

The dynamic behaviour of the obtained TF network was studied (under simulated hypoxic environment) using GINsim [16]. GINsim is a mathematical modelling tool which provides a Boolean/logical framework for efficient analysis of gene regulatory networks. In order to gain insights into semi-quantitative dynamics of the nodes of the constructed TF network without any prior knowledge of the kinetic parameters associated to the nodes, a multi-level Boolean framework was adopted. Two primary input requirements for model construction in GINsim correspond to – (i) values for basal and maximum expression levels of each node and (ii) logical rules governing activation of each node. The basal and maximum expression level of each node was obtained from the available microarray expression data over six time points under hypoxic stress [6]. The expression data was scaled and discretized in order to suit the format of GINsim (Table S6.1).

**Table S6.1:** Re-scaled values of expression levels (provided in Rustad et al. 2008) of 24 transcription factors (TFs). These TFs belong to the TF network that has been modeled (using a Boolean framework) in the present study. All gene expression values have been scaled to four (viz. 0, 1, 2, 3) discrete levels for ease of simulation of the Boolean model.

| **Transcription factor** | **Scaled expression** | | | | | |
| --- | --- | --- | --- | --- | --- | --- |
|  | **4 hour** | **8 hour** | **12 hour** | **1 day** | **4 days** | **7 days** |
| cmtR | 2 | 3 | 3 | 3 | 3 | 3 |
| csoR | 1 | 3 | 3 | 3 | 3 | 3 |
| devR | 3 | 3 | 2 | 2 | 1 | 1 |
| devS | 3 | 2 | 1 | 1 | 0 | 0 |
| hrcA | 1 | 2 | 2 | 1 | 2 | 2 |
| kmtR | 2 | 2 | 2 | 2 | 2 | 2 |
| kstR | 1 | 2 | 3 | 2 | 3 | 2 |
| lsr2 | 0 | 0 | 1 | 1 | 3 | 3 |
| phoP | 1 | 1 | 1 | 2 | 2 | 2 |
| Rv0081 | 2 | 2 | 1 | 1 | 1 | 1 |
| Rv0324 | 1 | 1 | 2 | 2 | 2 | 2 |
| Rv0348 | 1 | 2 | 3 | 3 | 3 | 3 |
| Rv0474 | 1 | 2 | 2 | 3 | 3 | 3 |
| Rv0494 | 1 | 1 | 2 | 1 | 2 | 2 |
| Rv0653c | 1 | 1 | 1 | 1 | 2 | 2 |
| Rv0767c | 1 | 1 | 2 | 3 | 3 | 3 |
| Rv1255c | 0 | 0 | 0 | 1 | 3 | 2 |
| Rv1985c | 1 | 2 | 2 | 2 | 3 | 3 |
| Rv1990c | 1 | 3 | 3 | 2 | 3 | 3 |
| Rv2011c | 1 | 2 | 3 | 2 | 2 | 3 |
| Rv2021c | 0 | 0 | 1 | 2 | 3 | 3 |
| Rv2034 | 1 | 2 | 3 | 3 | 3 | 3 |
| Rv2324 | 0 | 2 | 2 | 2 | 3 | 2 |
| whiB4 | 1 | 2 | 2 | 2 | 2 | 3 |

The logical rule for activation of the TFs were evaluated from the regulatory interaction collated in the current study and microarray expression data obtained from the study by Rustad and colleagues [6]. The logical rule for a particular node was represented by its regulators joined with logical operators like ‘AND’, ‘OR’ and ‘NOT’, as mentioned in previous literatures [17,18]. For example, if a node ‘X’ is positively regulated by two nodes R1 and R2, the logical rule for activation of ‘X’ can be written in either of the two possible ways – (a) ‘R1 AND R2’, (b) ‘R1 OR R2’. An ‘AND’ operator was selected when, R1 and R2 was observed to have positive interaction (from the regulatory network assembled in the current study) and a correlated expression pattern. In case where this criteria was not satisfied, an ‘OR’ operator was used in the logical rule. The logical rule assigned to each node of the network is listed in (Table S6.2). Thus, in the present multi-level Boolean model, the nodes of the network could acquire four discrete values (0 to 3) depending on the values of its regulators. The negative regulations were represented by a ‘NOT’ operator in the logical rules, which allowed reduction in the expression level of a particular node in presence of its negative regulator(s). It may be noted that for some of the nodes the effects of its repressors were ignored as expression levels of both the target node and its repressor(s) were observed to increase with time under hypoxic environment. For example, the node whiB4 was observed to be negatively regulated by another node Rv2011c in the TF network collated from literature (Figure S6.1). In contrast, the expression levels of both whiB4 and Rv2011c were shown to be increasing in the microarray expression data. Such discrepancies may arise due to methodological differences in the corresponding studies. Thus, such regulatory effects were excluded while constructing the Boolean model.

**Table S6.2:** Logical rules governing expression of each node of the TF (transcription factor) network controlling hypoxia.

| **Node** | **Logical rule** | **Reference** |
| --- | --- | --- |
| CmtR | (Lsr2 OR Rv0081 OR Rv0676c OR CmtR) AND (NOT Rv0324) | Minch et al. 2014 |
| CsoR | (Rv0081 OR CsoR) AND (NOT Rv2324) | Minch et al. 2014 |
| DevR | DevS OR (PhoP AND Rv2034) OR (DevR AND (DevS OR (PhoP AND Rv2034))) | Guo et al. 2009, Honaker et al. 2009, Gao et al. 2012, Galagan et al. 2013, Minch et al. 2014 |
| DevS | DevR | Guo et al. 2009, Galagan et al. 2013 |
| HrcA | Rv0324 | Minch et al. 2014 |
| KmtR | KmtR AND (NOT Rv2034) | Minch et al. 2014 |
| KstR | (Rv0348 OR Rv2324 OR KstR OR (Lsr2 AND Rv0324)) AND (NOT Rv0081) | Minch et al. 2014 |
| Lsr2 | (Rv1985 OR CmtR OR Rv2034 OR KstR OR Lsr2) AND (NOT(PhoP OR Rv0324 OR Rv0474)) | Galagan et al. 2013, Minch et al. 2014 |
| PhoP | (WhiB4 OR Rv0081 OR (Lsr2 AND Rv2034)) AND (NOT Rv2011) | Gupta et al. 2006, Gao et al. 2012, Galagan et al. 2013, Minch et al. 2014 |
| Rv0081 | DevR AND (NOT (Rv1990c OR Rv0081)) | He et al. 2011, Galagan et al. 2013 |
| Rv0324 | (Rv0324 OR (Lsr2 AND Rv1985c)) AND (NOT Rv0081) | Galagan et al. 2013, Minch et al. 2014 |
| Rv0348 | Rv0081 AND (NOT (Rv0324 OR Rv1985c OR Rv2034)) | Minch et al. 2014 |
| Rv0474 | (Rv1985c OR Rv0081 OR Rv0474) AND (NOT CmtR) | Minch et al. 2014 |
| Rv0494 | (Rv0081 OR Rv0494) AND (NOT Rv0767c) | Minch et al. 2014 |
| Rv0653c | Lsr2 OR Rv0081 OR Rv0653c | Minch et al. 2014 |
| Rv0767c | External or internal regulators | - |
| Rv1255c | NOT Rv1990c | Minch et al. 2014 |
| Rv1985c | (Rv0081 OR Rv1985c) AND (NOT (DevR OR CmtR OR Rv0324)) | Minch et al. 2014 |
| Rv1990c | Lsr2 OR Rv0081 OR Rv1990c | Minch et al. 2014 |
| Rv2011c | Rv2011c | Minch et al. 2014 |
| Rv2021c | Rv0081 | Galagan et al. 2013 |
| Rv2034 | Rv0081 AND (NOT DevR) | Minch et al. 2014 |
| Rv2324 | External or internal regulators | - |
| WhiB4 | Rv0081 AND (Not Rv2011c) | Minch et al. 2014 |
| External or internal regulators | Input node | - |

The constructed multi-level Boolean model was then simulated under synchronous mode in GINsim. Simulation results were validated based on available gene expression data [6]. While simulating the model, the initial values of the variables were assigned according to the scaled expression at the first time point (i.e. 4 hours) (Table S6.1).

1. **Construction and Boolean modelling of the transcription factor (TF)-gene interaction network in *M. tuberculosis* H37Rv (Mtb)**

The ‘target’ genes (referred here as the ‘downstream’ genes) which are regulated by each of the 24 transcription factors in the TF network were identified based on the TF-gene pairs collated from literature (Additional File 7). Thus, an extended gene regulatory network (GRN) was constructed. This GRN was explored using a Boolean model in GINsim [16]. Given the scarcity of experimental data, the regulation of the downstream genes was modeled following a binary framework, wherein, each node of the network was assigned a value of either ‘one’ or ‘zero’ representing ‘ON’ and ‘OFF’ states respectively. Also, values for basal and maximum expression levels of every node were kept as ‘zero’ and ‘one’ respectively. The logical rule for activation of each node was formulated based on the regulatory effects on the particular node. The logical rule for activation of a node ‘X’ having two positive regulators ‘R1’ and ‘R2’ was written as either ‘R1 AND R2’ or ‘R1 OR R2’, as mentioned in previous literatures [17,18]. An ‘AND’ operator was used when R1 and R2 were observed to have positive interaction and an ‘OR’ operator was used otherwise. Simulation of the model was performed mimicking wild type as well as deletion mutants pertaining to each of the 24 TFs. The *in silico* deletion mutants were simulated by keeping value of the corresponding TF as ‘zero’ throughout the simulation. For each case, first, the multi-level model of the TF network was simulated and steady state values corresponding to the 24 TFs were obtained (as discussed earlier). These steady state values were used to initialize the TFs for subsequent simulation of the (binary) Boolean model pertaining to the extended GRN. While simulating this model, the initial values of the downstream genes were set to ‘zero’. From each simulation, the list of active target genes (ON state) at steady state was obtained for further pathway enrichment (GO enrichment) analysis.

**Gene Ontology (GO) Enrichment Analysis**

Since a comprehensive list of GO annotations for Mtb proteins was not available from public repositories (or literature), a sequence homology based mapping between Mtb proteins and all bacterial entries having GO annotations from the Uniprot database ([www.uniprot.org/](http://www.uniprot.org/)) was performed using Blastp. The sequence homology search was done using highly stringent filtering criteria: e-value cut-off: 0.00001, coverage cut-off: 90% and identity cut-off: 90%). The GO terms associated to the best-hits from Uniprot database was used to annotate the query Mtb proteins. The hierarchy information obtained from the GO (Gene Ontology) Consortium database [9] was subsequently utilized to construct the GO hierarchy tree(s) for the annotated Mtb proteins. Following this procedure, GO annotations for ~75% of the Mtb proteins could be obtained. This is significantly higher than the annotations (~25% of Mtb proteins) available in the GO (Gene Ontology) Consortium database. The enrichment analysis, for any given set of Mtb proteins, was subsequently performed using an in-house script and GO annotations obtained through the above mentioned process. Pathways/ processes having a two-fold change in enrichment value with a p-value cut-off of 0.05 (Fisher exact test) and comprising of at least two proteins were considered to be enriched.

While performing the GO enrichment analysis of the Mtb proteins which were involved in HPIs with humans, the said set of Mtb proteins along with their 1^st^ and 2^nd^ degree neighbours were considered. The neighborhood information was collected from a filtered Mtb background PPI network [19]. Gene expression data obtained from microarray datasets [20,21] were used to ascertain up-/down-regulated pathways/ processes. The gene expression profile constituted of expression data pertaining to 1 hour, 4 hours, 18 hours and 24 hours time-points post infection. The gene expression values were divided into two sets: (a) early response (cumulating expression data collected before 18 hrs) and (b) late response (cumulating expression values collected at 18hrs and onwards). Overall perturbation profiles for a gene/protein at the early and late time points were calculated considering a consensus of its expressions during the constituent time points. A gene was tagged either as 'up' (resembling up-regulation), 'down' (resembling down-regulation), or '0' (resembling no-perturbation), depending on the number of times it was observed to be up/down-regulated, in the early and the late response sets. An enriched GO Biological process/pathway was inferred to be activated/enhanced (during either the early/late response stage) if a majority of the perturbed genes (considered for the GO enrichment study) contributing towards the pathway were up-regulated. Similarly, a majority of the contributing genes being down-regulated signifies a suppressed/attenuated biological process. In both these cases, the modulation of a biological pathway/ process was inferred only if a minimum of 30% observations (genes involved in the process being up/down-regulated during early and late stages of infection) were in consensus.

In order to gain additional insights into the criticality of the regulators (TFs) under hypoxic environment, the stable states obtained through Boolean model simulation (of the extended GRN) were further subjected to GO enrichment analysis. GO enrichment was performed on the gene sets observed to be ‘ON’/ active in the stable states corresponding to wild type as well as the simulated mutants. Subsequently, the result for each mutant was compared against that of the wild type to infer the variations in biochemical activities in the mutants.

**References**

1. Abomoelak B, Hoye EA, Chi J, Marcus SA, Laval F, Bannantine JP, et al. mosR, a novel transcriptional regulator of hypoxia and virulence in Mycobacterium tuberculosis. J Bacteriol. 2009;191:5941–52.

2. Gideon HP, Wilkinson KA, Rustad TR, Oni T, Guio H, Kozak RA, et al. Hypoxia induces an immunodominant target of tuberculosis specific T cells absent from common BCG vaccines. PLoS Pathog. 2010;6:e1001237.

3. Honaker RW, Leistikow RL, Bartek IL, Voskuil MI. Unique roles of DosT and DosS in DosR regulon induction and Mycobacterium tuberculosis dormancy. Infect Immun. 2009;77:3258–63.

4. Park H-D, Guinn KM, Harrell MI, Liao R, Voskuil MI, Tompa M, et al. Rv3133c/dosR is a transcription factor that mediates the hypoxic response of Mycobacterium tuberculosis. Mol Microbiol. 2003;48:833–43.

5. Rosenkrands I, Slayden RA, Crawford J, Aagaard C, Barry CE, Andersen P. Hypoxic response of Mycobacterium tuberculosis studied by metabolic labeling and proteome analysis of cellular and extracellular proteins. J Bacteriol. 2002;184:3485–91.

6. Rustad TR, Harrell MI, Liao R, Sherman DR. The enduring hypoxic response of Mycobacterium tuberculosis. PLoS ONE. 2008;3:e1502.

7. Sherman DR, Voskuil M, Schnappinger D, Liao R, Harrell MI, Schoolnik GK. Regulation of the Mycobacterium tuberculosis hypoxic response gene encoding alpha -crystallin. Proc Natl Acad Sci USA. 2001;98:7534–9.

8. Steyn AJC, Collins DM, Hondalus MK, Jacobs WR, Kawakami RP, Bloom BR. Mycobacterium tuberculosis WhiB3 interacts with RpoV to affect host survival but is dispensable for in vivo growth. Proc Natl Acad Sci USA. 2002;99:3147–52.

9. Ashburner M, Ball CA, Blake JA, Botstein D, Butler H, Cherry JM, et al. Gene ontology: tool for the unification of biology. The Gene Ontology Consortium. Nat Genet. 2000;25:25–9.

10. Galagan JE, Minch K, Peterson M, Lyubetskaya A, Azizi E, Sweet L, et al. The Mycobacterium tuberculosis regulatory network and hypoxia. Nature. 2013;499:178–83.

11. Gao C, Yang M, He Z-G. Characterization of a novel ArsR-like regulator encoded by Rv2034 in Mycobacterium tuberculosis. PLoS ONE. 2012;7:e36255.

12. Guo M, Feng H, Zhang J, Wang W, Wang Y, Li Y, et al. Dissecting transcription regulatory pathways through a new bacterial one-hybrid reporter system. Genome Res. 2009;19:1301–8.

13. Gupta S, Sinha A, Sarkar D. Transcriptional autoregulation by Mycobacterium tuberculosis PhoP involves recognition of novel direct repeat sequences in the regulatory region of the promoter. FEBS Lett. 2006;580:5328–38.

14. He H, Bretl DJ, Penoske RM, Anderson DM, Zahrt TC. Components of the Rv0081-Rv0088 locus, which encodes a predicted formate hydrogenlyase complex, are coregulated by Rv0081, MprA, and DosR in Mycobacterium tuberculosis. J Bacteriol. 2011;193:5105–18.

15. Minch KJ, Rustad TR, Peterson EJR, Winkler J, Reiss DJ, Ma S, et al. The DNA-binding network of Mycobacterium tuberculosis. Nature Communications. 2015;6:5829.

16. Gonzalez AG, Naldi A, Sánchez L, Thieffry D, Chaouiya C. GINsim: a software suite for the qualitative modelling, simulation and analysis of regulatory networks. BioSystems. 2006;84:91–100.

17. Das C, Dutta A, Rajasingh H, Mande SS. Understanding the sequential activation of Type III and Type VI Secretion Systems in Salmonella typhimurium using Boolean modeling. Gut Pathog. 2013;5:28.

18. Hegde SR, Rajasingh H, Das C, Mande SS, Mande SC. Understanding Communication Signals during Mycobacterial Latency through Predicted Genome-Wide Protein Interactions and Boolean Modeling. PLOS ONE. 2012;7:e33893.

19. Karim AF, Chandra P, Chopra A, Siddiqui Z, Bhaskar A, Singh A, et al. Express path analysis identifies a tyrosine kinase Src-centric network regulating divergent host responses to Mycobacterium tuberculosis infection. J Biol Chem. 2011;286:40307–19.

20. Edgar R, Domrachev M, Lash AE. Gene Expression Omnibus: NCBI gene expression and hybridization array data repository. Nucleic Acids Res. 2002;30:207–10.

21. Witney AA, Waldron DE, Brooks LA, Tyler RH, Withers M, Stoker NG, et al. BμG@Sbase—a microbial gene expression and comparative genomic database. Nucleic Acids Res. 2012;40:D605–9.
