# Supplementary material for: Understanding the role of interactions between host and Mycobacterium tuberculosis under hypoxic condition: an in silico approach
Source: BMC Genomics. 2018 Jul 27;19:555. doi: 10.1186/s12864-018-4947-8 (PMC6064076; doi:10.1186/s12864-018-4947-8)
Supplement: Supplementary file 8 — Comparison of results of the multi-level Boolean model simulation with experimentally obtained gene expression data. (DOCX 71 kb) [file 12864_2018_4947_MOESM8_ESM.docx]

Additional File 8: Comparison of results of the multi-level Boolean model simulation with experimentally obtained gene expression data.


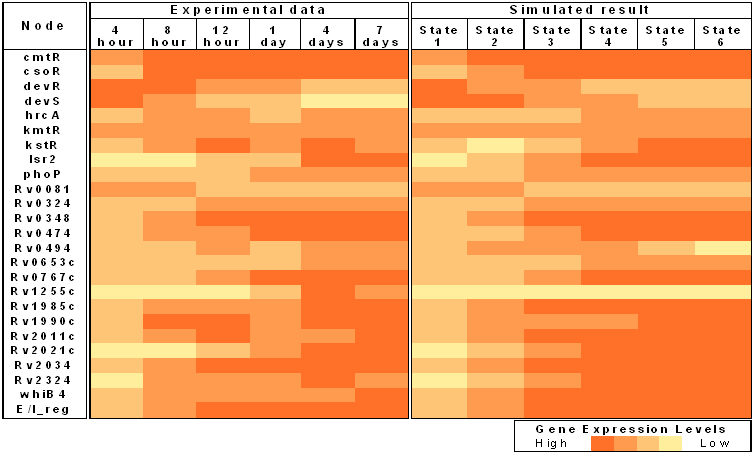


Figure S8.1: Comparison of results of the multi-level Boolean model simulation of Mtb hypoxia gene regulatory network with experimentally obtained gene expression data. Gene expression data from the experiments are represented in 4 discrete levels for ease of comparison.
