# Supplementary material for: Understanding the role of interactions between host and Mycobacterium tuberculosis under hypoxic condition: an in silico approach
Source: BMC Genomics. 2018 Jul 27;19:555. doi: 10.1186/s12864-018-4947-8 (PMC6064076; doi:10.1186/s12864-018-4947-8)
Supplement: Supplementary file 9 — Results of the analysis on gene regulatory network (GRN) controlling hypoxic response in M. tuberculosis H37Rv. (DOCX 14 kb) [file 12864_2018_4947_MOESM9_ESM.docx]

**Additional File 9: Results of the analysis on gene regulatory network (GRN) controlling hypoxic response in *M. tuberculosis* H37Rv (Mtb)**

The transcription factor (TF) network (comprising of 24 TFs belonging to the *dosR* and EHR regulons) involved in gene regulatory events during Mtb hypoxic response was investigated through Boolean modelling (details in Additional File 6). The multi-level Boolean model constructed in the current study was simulated with an initial condition that represents the biological state (gene expression levels) at the beginning of hypoxic stress. The simulation progressed through four intermediate states to reach a final ‘stable state’, indicating the gene expression levels that would be expected in a mycobacterial cell under hypoxic stress (Additional File 8). In order to validate the model, the stable state (and the intermediate states) obtained from the simulation were compared to the experimentally observed gene expression values (see Additional File 6). Interestingly, for over 70% of the total TFs (nodes) in the network, the stable state values obtained from simulation were observed to be in coherence with the experimental values (Additional File 8). Apart from capturing the gradual changes in pattern of expression for most of the TFs (across time-points or intermediate states), the simulation results indicated oscillated expression of two of the TFs (*kstR* and Rv0494), which were in line with earlier experimental observations [1]. Overall, these observations indicate that, the constructed Boolean model is fairly able to capture the underlying regulatory mechanism of the TF network involved in hypoxic response of Mtb.

**References**

1. Rustad TR, Harrell MI, Liao R, Sherman DR. The enduring hypoxic response of Mycobacterium tuberculosis. PLoS ONE. 2008;3:e1502.
