# Supplementary material for: Understanding the role of interactions between host and Mycobacterium tuberculosis under hypoxic condition: an in silico approach
Source: BMC Genomics. 2018 Jul 27;19:555. doi: 10.1186/s12864-018-4947-8 (PMC6064076; doi:10.1186/s12864-018-4947-8)
Supplement: Supplementary file 10 — Details of the adopted method and corresponding results of Flux Balance Analysis (FBA) of M. tuberculosis H37Rv metabolism during hypoxia. (DOCX 27 kb) [file 12864_2018_4947_MOESM10_ESM.docx]

**Additional File 10: Details of adopted method and results of Flux Balance Analysis (FBA) of *M. tuberculosis* H37Rv (Mtb) metabolism during hypoxia**

The available whole genome metabolic network of Mtb, iNJ661 [1] was used in the present study. Notably, lipids are known to be preferred (over carbohydrates) as source of carbon (for energy) by Mtb for sustenance inside the host cells [2]. Given that the iNJ661 metabolic model lacked several enzymes (and reactions) pertaining to lipid metabolism (especially cholesterol metabolism), the model was manually curated for ‘gaps’ in lipid metabolism. These gaps were subsequently filled using information from public databases [3,4]. The final iNJ661 model (appended with additional enzymes and reactions for lipid metabolism) was utilized for the FBA simulation studies.

For this purpose, a previously published metabolic model of *M. tuberculosis* H37Rv (iNJ661 model) was chosen [1]. It may be noted that the iNJ661 model lacks some components of lipid metabolism, especially those pertaining to cholesterol metabolism. Given that, fatty acids and cholesterol have been proposed to be the preferred carbon sources for Mtb during its intra-cellular survival, enzymes and reactions for cholesterol degradation pathway were added to the iNJ661 model. The updated iNJ661 model (details in Additional File 11) was simulated for growth (with biomass as the objective function) using M9 minimal media and varying carbon sources. In the first instance, the model was simulated with glucose as the principal carbon source, thereby mimicking an aerobic broth culture. Subsequently, the model was simulated under minimal oxygen conditions to mimic hypoxia. In this case, the simulation was performed in the absence of glucose (and citrate). As expected, while glucose was utilized as the main carbon source by the simulated Mtb cells in the first instance, fatty acids and cholesterol were utilized for growth in the simulated hypoxic condition. The Mtb bacilli were seen to grow around 19% slower under hypoxia (and glucose limited) condition, as compared to aerobic steady state conditions. This observation is in line with the data presented in one of the recent studies on the growth rates of clinical isolates of *M. tuberculosis* under aerobic and hypoxic conditions [5].

Overall, 60 reactions (catalyzed by 78 enzymes) were found to be significantly perturbed during hypoxic metabolism (with respect to the simulation mimicking aerobic growth), wherein more than a 2-fold change in flux was observed (Additional File 12). The average flux through the reactions constituting each of the metabolic pathways was also computed (Table S11.1). Primarily, metabolic pathways constituting the central carbon metabolism, viz., citric acid cycle (TCA cycle), pyruvate metabolism and glycolysis, along with nucleotide metabolism (mostly purine metabolism) were seen to be perturbed during hypoxia. In addition, fluxes through metabolic pathways corresponding to certain amino acids like arginine, proline, glycine, serine and threonine were found to be altered. Furthermore, redox reactions were also observed to carry differential flux under varying simulation conditions.

**Table S11.1:** Average difference in flux (mM/gDW/hr) flowing through various pathways in *M. tuberculosis* H37Rv (Mtb) under aerobic and hypoxia condition

| **Pathways** | **Average_Flux_Difference** |
| --- | --- |
| Citric Acid Cycle | 17.75123767 |
| Pyruvate Metabolism | 17.15896988 |
| Purine Metabolism | 11.86777682 |
| Glycolysis | 3.438354579 |
| Redox Metabolism | 2.367983841 |
| Glutamate Metabolism | 1.091685581 |
| Arginine and Proline Metabolism | 0.597599601 |
| Glycine, Serine, and Threonine Metabolism | 0.521508133 |
| Fatty Acid Metabolism | 0.392304824 |
| Folate Metabolism | 0.29554436 |
| Other Amino Acid Metabolism | 0.295423453 |
| Cofactor Metabolism | 0.283975597 |
| Pentose Phosphate Pathway | 0.26135935 |
| Pyrimidine Metabolism | 0.229401304 |
| Valine, Leucine, and Isoleucine Metabolism | 0.166814256 |
| Alanine and Aspartate Metabolism | 0.122640506 |
| Sugar Metabolism | 0.077438428 |
| Phenylalanine Tyrosine Tryptophan Metabolism | 0.057086373 |
| Methionine Metabolism | 0.056746966 |
| Membrane Metabolism | 0.04257617 |
| Lysine Metabolism | 0.041797741 |
| Cysteine Metabolism | 0.035131967 |
| Polyprenyl Metabolism | 0.026495451 |
| Histidine Metabolism | 0.022610454 |
| Nucleotide Sugar Metabolism | 0.020842995 |
| Peptidoglycan Metabolism | 0.016307168 |
| Pantothenate and CoA Metabolism | 7.51E-14 |
| Porphyrin Metabolism | 1.09E-29 |

It was interesting to note that under hypoxic condition, oxaloacetate (OAA) was converted to malate through a reaction catalyzed by malate dehydrogenase (MDH). This indicated a reversal in the direction of the reaction flux when compared to the simulation mimicking aerobic condition. Furthermore, an influx of CO_2_ into the mycobacterial cell was observed during hypoxia. Previous studies have indicated that CO_2_ may be assimilated through anaplerotic pathways to produce OAA which may then be transformed into citrate through the TCA cycle in glucose limited conditions [6,7]. The observations from the current study also suggest an increased flux through the reactions surrounding the OAA node in the Mtb metabolic network during hypoxia. Furthermore, a reversal in flux direction was observed through three reactions in the glycolytic pathway during hypoxia. These included reactions catalyzed by FBA (fructose-bisphosphate aldolase), PGI (glucose-6-phosphate isomerase), and PPGK (polyphosphate glucokinase).

Another interesting observation pertained to the apparent inability of Mtb to synthesize vitamin B12 under hypoxia, indicating that the bacterium is probably dependent on vitamins from the host for its survival. Some of the other reactions where the flow of flux was observed to be reversed during hypoxia included those catalyzed by ARI (arabinose ribose isomerase), THRD (L-threonine deaminse), NDPK6 (nucleoside-diphosphate kinase), and SHSL (O-succinylhomoserine lyase). In summary, the FBA study suggests that the metabolism of Mtb undergoes a significant re-routing during hypoxia (anaerobic) as compared to aerobic conditions. In addition to a lowered growth rate and utilization of different carbon sources for survival, this change is characterized by reversal in the flow of flux through several key reactions in the central carbon metabolism.

**References**

1. Jamshidi N, Palsson BØ. Investigating the metabolic capabilities of Mycobacterium tuberculosis H37Rv using the in silico strain iNJ 661 and proposing alternative drug targets. BMC Systems Biology. 2007;1:26.

2. Muñoz-Elías EJ, McKinney JD. Mycobacterium tuberculosis isocitrate lyases 1 and 2 are jointly required for in vivo growth and virulence. Nat Med. 2005;11:638–44.

3. Kanehisa M, Furumichi M, Tanabe M, Sato Y, Morishima K. KEGG: new perspectives on genomes, pathways, diseases and drugs. Nucleic Acids Res. 2017;45:D353–61.

4. King ZA, Lu J, Dräger A, Miller P, Federowicz S, Lerman JA, et al. BiGG Models: A platform for integrating, standardizing and sharing genome-scale models. Nucleic Acids Res. 2016;44:D515-522.

5. Liu Z, Gao Y, Yang H, Bao H, Qin L, Zhu C, et al. Impact of Hypoxia on Drug Resistance and Growth Characteristics of Mycobacterium tuberculosis Clinical Isolates. PLoS ONE. 2016;11:e0166052.

6. Baek S-H, Li AH, Sassetti CM. Metabolic regulation of mycobacterial growth and antibiotic sensitivity. PLoS Biol. 2011;9:e1001065.

7. Machová I, Snášel J, Zimmermann M, Laubitz D, Plocinski P, Oehlmann W, et al. Mycobacterium tuberculosis Phosphoenolpyruvate Carboxykinase Is Regulated by Redox Mechanisms and Interaction with Thioredoxin. J Biol Chem. 2014;289:13066–78.
