# Supplementary material for: Understanding the role of interactions between host and Mycobacterium tuberculosis under hypoxic condition: an in silico approach
Source: BMC Genomics. 2018 Jul 27;19:555. doi: 10.1186/s12864-018-4947-8 (PMC6064076; doi:10.1186/s12864-018-4947-8)
Supplement: Supplementary file 15 — Details of the methodology adopted for identifying the shortest-paths among (A) the HPI-network, (B) the M. tuberculosis H37Rv hypoxic- gene regulatory network, and (C) the hypoxic-metabolism network of M. tuberculosis H37Rv. (DOCX 19 kb) [file 12864_2018_4947_MOESM15_ESM.docx]

**Additional File 15: Identification of shortest-paths**

In order to recognize the paths carrying information between the three modules (during intra-cellular survival), viz., (A) the HPI-network, (B) the Mtb hypoxic-GRN, and (C) the hypoxic-metabolism network of Mtb, the shortest-path analysis was performed. For this purpose, the Mtb background PPI network was obtained from the STRING database [1] and filtered for a combined score cut-off of greater than (or equal to) 900. All paths connecting (i) 28 (out of 30) Mtb proteins involved in HPI with the host, (ii) 30 TFs from the Mtb hypoxic-GRN, and (iii) 78 Mtb enzymes, the reactions catalyzed by which were maximally perturbed during hypoxia), were first obtained using CompNet [2]. All identified paths were subsequently filtered for a maximal path-length of five. Paths shorter (or equal) to a path-length of five, were then checked for perturbed expression of the constituent genes [3–5]. Paths, wherein, at least 50% of the genes were observed to be perturbed were considered to be shortest-paths carrying information between the three modules.

The number of identified (perturbed) shortest-paths connecting the modules (A) to (B), (A) to (C), and (B) to (C) were 2554, 13151, and 4035 respectively. Furthermore, to ascertain that the number of identified (perturbed) shortest-paths between any two modules were statistically significant; a t-test was performed. In this test, for a given pair of modules, two sets of proteins equal to the size of the two modules were drawn from the (filtered) Mtb background network and (perturbed) shortest-paths (connecting the two identified sets of protein) were identified (as mentioned earlier). Using 100 iterations for each pair of the studied modules, the t-test ascertained that the observed shortest-paths connecting these modules were indeed significant with a p-value of lesser than 0.001.

**References**

1. Franceschini A, Szklarczyk D, Frankild S, Kuhn M, Simonovic M, Roth A, et al. STRING v9.1: protein-protein interaction networks, with increased coverage and integration. Nucleic Acids Res. 2013;41:D808–15.

2. Kuntal BK, Dutta A, Mande SS. CompNet: a GUI based tool for comparison of multiple biological interaction networks. BMC Bioinformatics. 2016;17:185.

3. Edgar R, Domrachev M, Lash AE. Gene Expression Omnibus: NCBI gene expression and hybridization array data repository. Nucleic Acids Res. 2002;30:207–10.

4. Karim AF, Chandra P, Chopra A, Siddiqui Z, Bhaskar A, Singh A, et al. Express path analysis identifies a tyrosine kinase Src-centric network regulating divergent host responses to Mycobacterium tuberculosis infection. J Biol Chem. 2011;286:40307–19.

5. Witney AA, Waldron DE, Brooks LA, Tyler RH, Withers M, Stoker NG, et al. BμG@Sbase—a microbial gene expression and comparative genomic database. Nucleic Acids Res. 2012;40:D605–9.
